# Arginase expression modulates nitric oxide production in *Leishmania (Leishmania) amazonensis*

**DOI:** 10.1371/journal.pone.0187186

**Published:** 2017-11-14

**Authors:** Stephanie Maia Acuña, Juliana Ide Aoki, Maria Fernanda Laranjeira-Silva, Ricardo Andrade Zampieri, Juliane Cristina Ribeiro Fernandes, Sandra Marcia Muxel, Lucile Maria Floeter-Winter

**Affiliations:** Departamento de Fisiologia, Instituto de Biociências, Universidade de São Paulo, São Paulo, Brazil; University of Rome, ITALY

## Abstract

**Background:**

Arginase is an enzyme that converts L-arginine to urea and L-ornithine, an essential substrate for the polyamine pathway supporting *Leishmania (Leishmania) amazonensis* replication and its survival in the mammalian host. L-arginine is also the substrate of macrophage nitric oxide synthase 2 (NOS2) to produce nitric oxide (NO) that kills the parasite. This competition can define the fate of *Leishmania* infection.

**Methodology/Principal findings:**

The transcriptomic profiling identified a family of oxidoreductases in *L*. *(L*.*) amazonensis* wild-type (*La*-WT) and *L*. *(L*.*) amazonensis* arginase knockout (*La*-arg^-^) promastigotes and axenic amastigotes. We highlighted the identification of an oxidoreductase that could act as nitric oxide synthase-like (NOS-like), due to the following evidences: conserved domain composition, the participation of NO production during the time course of promastigotes growth and during the axenic amastigotes differentiation, regulation dependence on arginase activity, as well as reduction of NO amount through the NOS activity inhibition. NO quantification was measured by DAF-FM labeling analysis in a flow cytometry.

**Conclusions/Significance:**

We described an arginase-dependent NOS-like activity in *L*. *(L*.*) amazonensis* and its role in the parasite growth. The increased detection of NO production in the mid-stationary and late-stationary growth phases of *La*-WT promastigotes could suggest that this production is an important factor to metacyclogenesis triggering. On the other hand, *La*-arg^-^ showed an earlier increase in NO production compared to *La*-WT, suggesting that NO production can be arginase-dependent. Interestingly, *La*-WT and *La-arg*^*-*^ axenic amastigotes produced higher levels of NO than those observed in promastigotes. As a conclusion, our work suggested that NOS-like is expressed in *Leishmania* in the stationary growth phase promastigotes and amastigotes, and could be correlated to metacyclogenesis and amastigotes growth in a dependent way to the internal pool of L-arginine and arginase activity.

## Introduction

Leishmaniasis is an important neglected tropical disease caused by the protozoa from *Leishmania* genus [1,2]. The infection caused by *L*. *(L*.*) amazonensis* can generate cutaneous and/or diffuse cutaneous manifestations [1,2]. This parasite has a dimorphic life cycle: the procyclic promastigote form can be found in the mid-gut of the phlebotomine sand fly; the metacyclic promastigote can be found in the insect foregut and stomodeal valve and corresponds to the infective form; and the amastigote form can be found into macrophages of the mammalian host [3,4]. *Leishmania* has a complex metabolic arsenal to enable its survival, replication and differentiation in different environments of both invertebrate and vertebrate hosts [3–5].

In *L*. *(L*.*) amazonensis* (*La*-WT), the L-arginine uptake is mediated by the amino acid permease 3 (AAP3) [6], then metabolized by arginase to produce urea and ornithine, being this last a substrate of polyamine pathway [7,8]. The availability of L-arginine and arginase activity is essential for the survival of *Leishmania* inside both invertebrate and mammalian hosts [9–12]. Additionally, *L*. *(L*.*) amazonensis* harbors NOS-like activity, producing NO [13–17]. L-arginine availability can interfere in the half-life of AAP3 transcripts, increasing the stability of the transporter and L-arginine uptake in *L*. *(L*.*) amazonensis* promastigotes [6]. The absence of arginase activity in *L*. *(L*.*) amazonensis* arginase knockout (*La*-arg^-^) leaded to an increase in L-arginine and citrulline levels and decrease in proline, ornithine and putrescine levels [18]. The availability of L-arginine and arginase influence the survival of Leishmania in axenic conditions and inside its mammalian host [9–12,19–22].

NOS is an oxidoreductase enzyme that catalyzes the conversion of L-arginine and molecular oxygen into L-citrulline and NO, using NADPH, heme, FAD, FMN and tetrahydro-L-biopterin (BH4) as cofactors, producing N^ω^-hydroxy-L-arginine (OH-arginine or NOHA), an intermediate product that is oxidized into citrulline with the release of NO [23–25]. In mammalian cells, the amplitude and concentration of NO are dependent on the NOS isoform activity and its cellular/tissue distribution [24–26]. Many NO functions have been described as directly dependent on its concentration in different tissues [26,27]. In mammals, NO regulates synapses (nNOS or NOS1), promotes the killing of pathogens (iNOS or NOS2) and modulates vascular smooth muscle contraction (eNOS or NOS3) [26], among other functions. In addition, other organisms such as plants can produce NO during germination and flowering [28].

NO production has also been described in Trypanosomatidae parasites. Paveto’s group (1995) purified a calcium-dependent NOS from *Trypanosoma cruzi* and demonstrated an L-arginine conversion to NO and L-citrulline [29], similar to neuronal-NOS (nNOS or NOS1) from mammalian cells [24]. Piacenza (2001) showed a NOS-dependent NO production and its role in suppressing *T*. *cruzi* apoptosis [30]. Pereira (2015) showed NOS activity, citrulline and NO production in *T*. *cruzi* and reduction in NO production during contact with the extracellular matrix of host cells [31]. *Leishmania* species also presents an enzyme that produces citrulline and NO [6,13,14,16–18,20]. The first demonstration of *Leishmania-*NOS-like protein was described by Basu (1997) for *L*. *(L*.*) donovani* [32]. Later, a NOS-like activity was also described for *L*. *(L*.*) amazonensis*, *L*. *(V*.*) braziliensis* and *L*. *(L*.*) infantum chagasi* [13–15,33]. A transcriptomic profiling of *L*. *(L*.) *mexicana* showed the presence of a NOS-like (LmxM.19.1450.1, EC 1.14.13.39), an enzyme with similar levels in promastigotes, and in both macrophage-derived and axenic amastigotes [34].

Based on RNA-seq data, here we identified a family of putative oxidoreductases in *La*-WT and *La*-arg^-^ differentially expressed when we compared *La*-WT vs. *La*-arg^-^ promastigotes and *La*-WT promastigotes vs. axenic amastigotes, as well a NOS-like gene expression, validated by qPCR. These findings indicated that NO production could be related to the parasite growth phase and to arginase activity. The quantification of NO production in the promastigote growth curve and in axenic amastigote forms of *La*-WT, *La*-*arg*^*-*^ and the arginase addback (*La-arg*^*-*^*/+ARG*) revealed increased detection in the mid- to late-phase of the parasite growth. The absence of arginase activity induced early NO production compared with *La*-WT. The inhibition of NOS activity reduced NO amount and the frequency of metacyclic forms. Axenic amastigotes produced around 10-fold increased amount of NO compared with *La*-WT promastigotes, but the absence of arginase activity reduced NO production in both forms. Altogether, these data indicated a possible biological role for NO in parasite differentiation signaling.

## Methods

### *Leishmania* culture

*La*-WT (MHOM/BR/1973/M2269) promastigotes were maintained in culture at 25°C by inoculating 5x10^5^ cells/mL in 10 mL of M199 medium, supplemented with 10% fetal bovine serum (FBS; Gibco, South America), 5 ppm hemin, 100 μM adenine, 100 U penicillin, 100 μg/mL streptomycin, 40 mM HEPES-NaOH and 12 mM NaHCO_3_, at pH 7.0, for a week-long culture at low passages (P1- 5). *La-arg*^*-*^ promastigotes were maintained in the same M199 medium supplemented, as described above, with addition of 30 μg/mL hygromycin B, 30 μg/mL puromycin (Sigma, St. Louis, MO, USA) and 50 μM putrescine (Sigma, St. Louis, MO, USA) [20]. *La-arg*^*-*^/+ARG was maintained in the same medium of *La-arg*^*-*^ with addition of 20 μg/mL phleomycin [20].

The growth curve was performed starting from an initial inoculum of 5x10^5^ cells/mL from stationary growth phase, and then the number of parasites was determined using a Coulter Z1 particle counter (Beckman, Fullerton, CA, USA), on days 3, 5, 7 and 9.

### Axenic amastigote differentiation

Mid-logarithmic growth phase promastigotes of *La-*WT, *La-arg*^*-*^ or *La-arg*^*-*^/+ARG (5x10^6^, each) were transferred to 10 mL of M199 medium supplemented as described above at pH 5.5 and maintained at 34°C. After 4 days of differentiation, amastigotes were flushed 5 times using a 22G syringe needle, washed twice with PBS (950 x g, 10 min, 4°C) and counted in Neubauer chamber [35,36].

### NOS/NO inhibition assays

For analysis of differentiation of procyclic to metacyclic promastigote forms, promastigotes of *La-*WT and *La-arg*^*-*^ at day 3 or 5 of culture were treated with 10 mM of ω-nitro-L-arginine methyl ester (*L*-*NAME*, NOS inhibitor) and/or 100 μM of 2-(4-Carboxyphenyl)-4,4,5,5-tetramethylimidazoline-1-oxyl-3-oxide (*cPTIO*, NO scavenger) for two days in M199 medium, as described above, at 25°C. Next, the frequency of metacyclic forms was analyzed by flow cytometry.

For analysis of NO production during amastigotes differentiation, mid-logarithmic growth phase promastigotes of *La-*WT and *La-arg*^*-*^ were differentiated to amastigotes as described above, with 10 mM of *L*-*NAME and/or* 100 μM of *cPTIO* at days 0 or day 2 of culture and at day 4 of culture NO production was analyzed.

### Total RNA isolation and library construction

Total RNA from *La*-WT and *La*-arg^-^ promastigotes and axenic amastigotes was isolated using TRIzol reagent (Life Technologies, Carlsbad, CA, USA), according to the manufacturer’s instructions. RNA samples were treated with DNase I (Thermo Scientific, Lithuania, EU), and the RNA concentration was determined by spectrophotometry (Nanodrop ND1000, Thermo Scientific, USA). In addition, the RNA integrity was evaluated using an Agilent 2100 Bioanalyzer and Pico Agilent RNA 6000 kit (Agilent Technologies, Santa Clara, CA, USA), according to the manufacturer’s instructions. Library preparations from three independent biological replicates were performed using Strand-specific TrueSeq RNA-seq Library Prep (Illumina), according to the manufacturer’s instructions.

### RNA-seq and data analysis

Paired-end reads (125 bp) were obtained using the Illumina HiSeq 2000 platform at the Norwegian Sequencing Centre at the University of Oslo. Trimmomatic was used to remove the Illumina adapter sequences [37]. The quality of the produced data was analyzed using FastQC by the Phred quality score [38]. Reads with Phred quality scores lower than 20 were discarded. The reads were aligned to the *L*. *mexicana* (MHOMGT2001U1103) genomic data obtained from TriTrypDB (www.tritryp.org) using TopHat [39,40]. Thereafter, read mapping was performed for transcript assembly using Cufflinks [41]. After assembly, the abundance of transcripts was calculated as the Fragments Per Kilobase of transcript per Million mapped reads (FPKM), which reflects the abundance of a transcript in the sample by normalization of the RNA length and the total read number [42]. Differentially expressed gene analysis was performed on four comparison pairs (*La*-WT promastigotes vs. *La*-arg^-^ promastigotes; *La*-WT axenic amastigotes vs. *La*-arg^-^ axenic amastigotes, *La*-WT promastigotes vs. *La*-WT axenic amastigotes; *La*-arg^-^ promastigotes vs. *La*-arg^-^ axenic amastigotes) [43].

### RT-qPCR

Reverse transcription was performed using 2 μg of total RNA as a template, reverse transcriptase and random primers (Revertaid H minus Reverse Transcriptase kit, Thermo-Scientific, Canada), according to the manufacturer’s instructions. Equal amounts of cDNA were assessed in duplicates in a total volume of 25 μL containing LuminoCt SYBR Green qPCR ReadyMix (SigmaAldrich, St Louis,MO, USA) and the following primers (200 nM): NOS-L_F 5´- acagcgactccaaaccttcc—3´, NOS-L_R 5´- gggacctgtttcagaggacg -3´, GAPDH_F 5´-tcaaggtcggtatcaacggc-3´, GAPDH_R 5´-tgcaccgtgtcgtacttcat-3´, amastin-like (LmxM.33.0960)_F, 5´-ggagcgctacttcagctatgga-3´, amastin-like_(LmxM.33.0960)_R, 5´-cggatcatcaataagacgatgttg-3´, amastin-like (LmxM.08.0800)_F 5´-agttcctcgcgtttctctttgt-3´, amastin-like (LmxM.08.0800)_R 5´ggcactgtgtactggcaaacc-3´, amastin-like (LmxM.08.0760)_F 5´-cggctgccttttgctgtact-3´ and amastin-like (LmxM.08.0760)_R 5´-cagacaacgcaagctgtgaca-3´. The mixture was incubated at 94°C for 5 min, followed by 40 cycles at 94°C for 30 s and 60°C for 30 s. Reactions were carried out using an PikoReal 96 RealTime PCR System (Thermo Scientfic, Finland). A negative control without reverse transcriptase was included in RT-qPCR assays to certify no contamination with DNA in RNA samples. A non-template control was included in all qPCR plates. The Fold Change of the target genes (*nosL* and *amastins-like*) and reference gene (*gapdh*) were quantified in three to six biological replicate samples, using a relative quantification.

### Quantification of NO production

Parasites were washed twice with PBS (1479 x g, 10 min, 4°C) and were incubated with 1 μM DAF-FM (4-amino-5methylamino-2’,7’-difluorofluorescein diacetate) (Life Technologies, Eugene, OR, USA) in PBS for 30 min at 25°C or 34°C for promastigotes and axenic amastigotes, respectively. Next, the cells were washed with PBS and were labeled with 1 ng/mL propidium iodide (PI) to analyze cell viability. Fluorescence acquisition was performed using a FACSCalibur (BD, Franklin Lakes, NJ, USA), and the collected data were analyzed using FlowJo Software (LLC, Ashland, OR, USA). The frequency of DAF-FM-positive cells and the mean fluorescence intensity (MFI) of DAF-FM-labeled cells (FL1 detector) were analyzed in viable cells (PI^-;^ FL2 detector) from 20,000 events, based in forward scatter (FSC) and side scatter (SSC) features.

### Statistical analysis

Statistical analysis was determined based on Student’s t-test, one-way ANOVA or Tukey’s comparison post-test, using the GraphPad Prism 6 Software (GraphPad Software, Inc., La Jolla, CA, USA) and considering p < 0.05 as significant.

## Results

### Oxidoreductases transcriptomic profiling of *La*-WT and *La*-arg^-^ promastigotes and axenic amastigotes

The transcriptomic profiling data of *La*-WT and *La-arg*^*-*^ promastigotes and axenic amastigotes are available on the NCBI BioProject under the accession number PRJNA380128 and Sequence Read Archive (SRA) under accession numbers SRX2661998 and SRX2661999 [43]. Based on this RNA-seq data, we identified oxidoreductases transcripts expression in the samples *La*-WT and *La-arg*^-^ promastigotes and axenic amastigotes (Tables 1–4).

**Table 1:** Oxidoreductase-like transcripts expression of *La*-WT and *La*-arg^-^ promastigotes.

ID	EC number	pro *La*-WT vs. pro *La-arg*^*-*^ Product description	Fold change
LmxM.03.0570	1.6.5.5	quinone oxidoreductase, putative	0.23
LmxM.05.0980	1.6.5.3	NADH-ubiquinone oxidoreductase, putative	1.05
LmxM.07.0600	1.5.5.1	electron transfer oxidoreductase, putative	0.88
LmxM.12.1130	1.3.1.42	NADH:flavin oxidoreductase/NADH oxidase, putative	0.93
LmxM.13.0720	1.6.5.5	oxidoreductase-like protein	0.75
LmxM.17.0270	1.6.5.3	NADH: ubiquinone oxidoreductase, putative	0.88
LmxM.18.1480	1.6.5.3	NADH: ubiquinone oxidoreductase, putative	1.08
LmxM.20.0120	no data	NADH: ubiquinone oxidoreductase, putative	0.69
LmxM.21.0420	1.18.1.6	NADH: adrenodoxin oxidoreductase, putative	0.60
LmxM.23.0670	no data	oxidoreductase-like protein	1.09
LmxM.23.0860	1.6.5.5	quinone oxidoreductase, putative	0.98
LmxM.23.1590	no data	oxidoreductase-like protein	0.74
LmxM.23.1600	no data	oxidoreductase-like protein	0.86
LmxM.28.0940	no data	oxidoreductase-like protein	0.51
LmxM.31.2210	no data	NADH: adrenodoxin oxidoreductase, putative	0.80
LmxM.32.1770	no data	oxidoreductase-like protein	0.79
LmxM.33.4320	no data	NADH: adrenodoxin oxidoreductase, putative	0.56
LmxM.36.3230	1.1.99.1	oxidoreductase-like protein	0.59
LmxM.36.4170	1.6.5.5	oxidoreductase-like protein	0.63
LmxM.36.5660	no data	FAD dependent oxidoreductase	0.87

Comparison of the oxidoreductases-orthologs transcripts between pro *La*-WT vs. pro *La*-arg^-^, adjusted for p < 0.05. pro: promastigote, *La*-WT: *L*. *(L*.*) amazonensis* wild-type, *La*-arg^-^: *L*. *(L*.*) amazonensis* arginase knockout.

**Table 2:** Oxidoreductase-like transcripts expression of *La*-WT and *La*-arg^-^ axenic amastigotes.

ID	EC number	ama *La-*WT vs. ama *La-arg*^*-*^ Product description	Fold change
LmxM.03.0570	1.6.5.5	quinone oxidoreductase, putative	1.08
LmxM.05.0980	1.6.5.3	NADH-ubiquinone oxidoreductase, putative	1.22
LmxM.07.0600	1.5.5.1	electron transfer oxidoreductase, putative	1.12
LmxM.12.1130	1.3.1.42	NADH:flavin oxidoreductase/NADH oxidase, putative	1.18
LmxM.13.0720	1.6.5.5	oxidoreductase-like protein	1.17
LmxM.17.0270	1.6.5.3	NADH: ubiquinone oxidoreductase, putative	1.00
LmxM.18.1480	1.6.5.3	NADH: ubiquinone oxidoreductase, putative	1.26
LmxM.20.0120	no data	NADH: ubiquinone oxidoreductase, putative	1.43
LmxM.21.0420	1.18.1.6	NADH: adrenodoxin oxidoreductase, putative	1.33
LmxM.23.0670	no data	oxidoreductase-like protein	0.94
LmxM.23.0860	1.6.5.5	quinone oxidoreductase, putative	1.07
LmxM.23.1590	no data	oxidoreductase-like protein	1.06
LmxM.23.1600	no data	oxidoreductase-like protein	0.59
LmxM.28.0940	no data	oxidoreductase-like protein	1.22
LmxM.31.2210	no data	NADH: adrenodoxin oxidoreductase, putative	0.67
LmxM.32.1770	no data	oxidoreductase-like protein	1.01
LmxM.33.4320	no data	NADH: adrenodoxin oxidoreductase, putative	0.88
LmxM.36.3230	1.1.99.1	oxidoreductase-like protein	1.26
LmxM.36.4170	1.6.5.5	oxidoreductase-like protein	0.60
LmxM.36.5660	no data	FAD dependent oxidoreductase	1.81

Comparison of the oxidoreductases-orthologs transcripts between ama *La*-WT vs. pro *La*-arg^-^, adjusted for p < 0.05. ama: axenic amastigotes, *La*-WT: *L*. *(L*.*) amazonensis* wild-type, *La*-arg^-^: *L*. *(L*.*) amazonensis* arginase knockout.

**Table 3:** Oxidoreductase-like transcripts expression of *La*-WT promastigotes and axenic amastigotes.

ID	EC number	pro *La-*WT vs. ama *La-*WT Product description	Fold change
LmxM.03.0570	1.6.5.5	quinone oxidoreductase, putative	0.48
LmxM.05.0980	1.6.5.3	NADH-ubiquinone oxidoreductase, putative	0.62
LmxM.07.0600	1.5.5.1	electron transfer oxidoreductase, putative	0.84
LmxM.12.1130	1.3.1.42	NADH:flavin oxidoreductase/NADH oxidase, putative	1.31
LmxM.13.0720	1.6.5.5	oxidoreductase-like protein	0.74
LmxM.17.0270	1.6.5.3	NADH: ubiquinone oxidoreductase, putative	0.85
LmxM.18.1480	1.6.5.3	NADH: ubiquinone oxidoreductase, putative	0.82
LmxM.20.0120	no data	NADH: ubiquinone oxidoreductase, putative	0.53
LmxM.21.0420	1.18.1.6	NADH: adrenodoxin oxidoreductase, putative	0.66
LmxM.23.0670	no data	oxidoreductase-like protein	0.91
LmxM.23.0860	1.6.5.5	quinone oxidoreductase, putative	0.78
LmxM.23.1590	no data	oxidoreductase-like protein	1.11
LmxM.23.1600	no data	oxidoreductase-like protein	0.99
LmxM.28.0940	no data	oxidoreductase-like protein	1.11
LmxM.31.2210	no data	NADH: adrenodoxin oxidoreductase, putative	1.64
LmxM.32.1770	no data	oxidoreductase-like protein	1.79
LmxM.33.4320	no data	NADH: adrenodoxin oxidoreductase, putative	0.81
LmxM.36.3230	1.1.99.1	oxidoreductase-like protein	0.76
LmxM.36.4170	1.6.5.5	oxidoreductase-like protein	1.60
LmxM.36.5660	no data	FAD dependent oxidoreductase	1.03

Comparison of the oxidoreductases-orthologs transcripts between pro *La*-WT vs. ama *La*-WT, adjusted for p < 0.05. pro: promastigotes, ama: axenic amastigotes, *La*-WT: *L*. *(L*.*) amazonensis* wild-type.

**Table 4:** Oxidoreductase-like transcripts expression of *La*-arg^-^ promastigotes and axenic amastigotes.

ID	EC number	pro *La-arg*^*-*^ vs. ama *La-arg*^*-*^ Product description	Fold change
LmxM.03.0570	1.6.5.5	quinone oxidoreductase, putative	2.27
LmxM.05.0980	1.6.5.3	NADH-ubiquinone oxidoreductase, putative	0.73
LmxM.07.0600	1.5.5.1	electron transfer oxidoreductase, putative	1.07
LmxM.12.1130	1.3.1.42	NADH:flavin oxidoreductase/NADH oxidase, putative	1.66
LmxM.13.0720	1.6.5.5	oxidoreductase-like protein	1.16
LmxM.17.0270	1.6.5.3	NADH: ubiquinone oxidoreductase, putative	0.97
LmxM.18.1480	1.6.5.3	NADH: ubiquinone oxidoreductase, putative	0.96
LmxM.20.0120	No data	NADH: ubiquinone oxidoreductase, putative	1.09
LmxM.21.0420	1.18.1.6	NADH: adrenodoxin oxidoreductase, putative	1.47
LmxM.23.0670	No data	oxidoreductase-like protein	0.79
LmxM.23.0860	1.6.5.5	quinone oxidoreductase, putative	0.86
LmxM.23.1590	No data	oxidoreductase-like protein	1.59
LmxM.23.1600	No data	oxidoreductase-like protein	0.68
LmxM.28.0940	No data	oxidoreductase-like protein	2.64
LmxM.31.2210	No data	NADH: adrenodoxin oxidoreductase, putative	1.37
LmxM.32.1770	No data	oxidoreductase-like protein	2.27
LmxM.33.4320	No data	NADH: adrenodoxin oxidoreductase, putative	1.27
LmxM.36.3230	1.1.99.1	oxidoreductase-like protein	1.62
LmxM.36.4170	1.6.5.5	oxidoreductase-like protein	1.52
LmxM.36.5660	No data	FAD dependent oxidoreductase	2.14

Comparison of the oxidoreductases-orthologs transcripts between pro *La*-arg^-^ vs. ama *La*-arg^-^, adjusted for p < 0.05. pro: promastigotes, ama: axenic amastigotes, *La*-WT: *L*. *(L*.*) amazonensis* wild-type, *La*-arg^-^: *L*. *(L*.*) amazonensis* arginase knockout.

**Fig 1:**
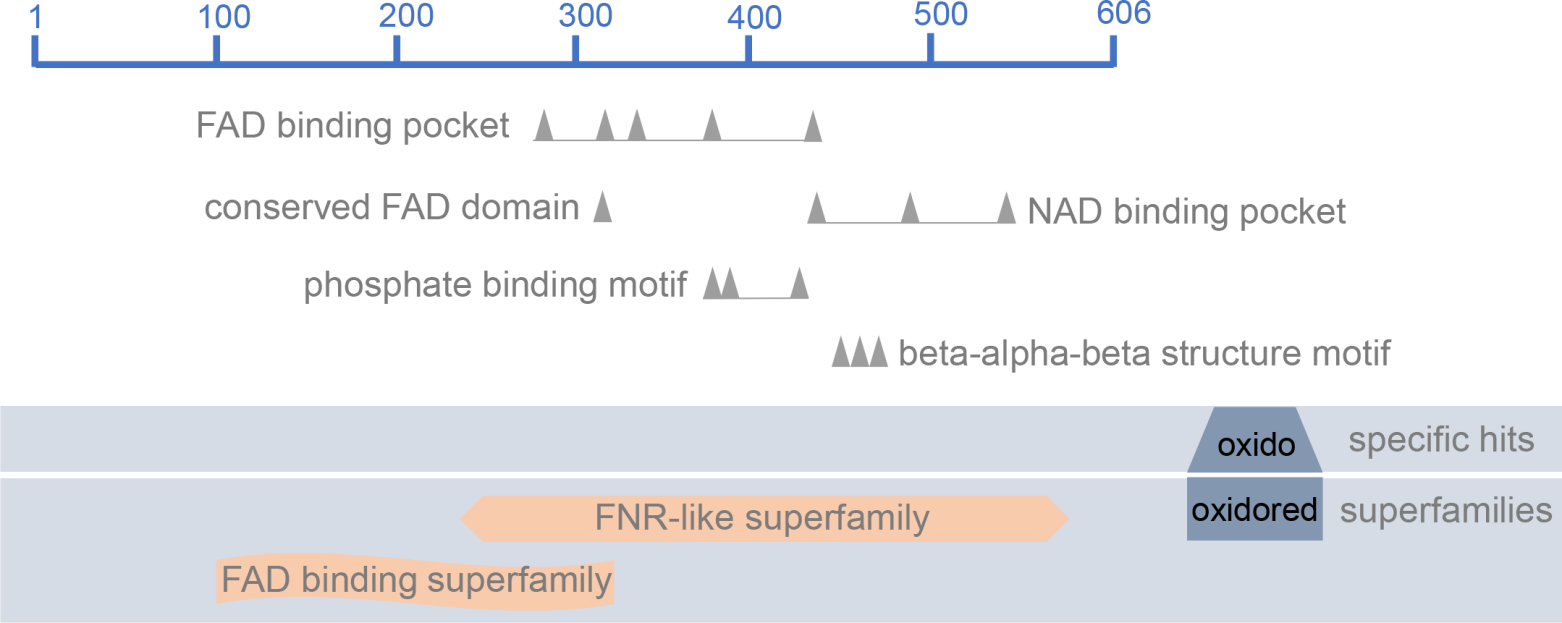
NOS-like graphical summary of conserved domains. Structure of the oxidoreductase synthase-like protein (LmxM.1950/EC 1.14.13.39) with the following conserved domains hits: ferredoxin reductase (FNR) binding domain, flavin adenine dinucleotide (FAD) binding pocket, conserved FAD binding motif, nicotinamide adenine dinucleotide (NAD) binding pocket, phosphate binding motif and beta-alpha-beta structure motif. The representation was based on *in silico* search of conserved domain of NCBI database.

The oxidoreductase synthase-like (LmxM.19.1450/ EC 1.14.13.39) was identified and appeared up-regulated in *La-arg*^-^ promastigotes when compared with *La*-WT promastigotes and in *La*-WT axenic amastigotes when compared with *La*-WT promastigotes, with fold-change 1.38 and 1.26, respectively. *In silico* search revealed the presence of the following oxidoreductase family domains: ferredoxin reductase (FNR)-like and flavin adenine dinucleotide (FAD) binding (Fig 1). In addition, previous data with metabolome fingerprints identified metabolic products such as NO and citrulline which could be produced by the enzyme (EC 1.14.13.39) [18].

*In silico* orthologs search revealed the presence of one gene copy per haploid genome in other *Leishmania* species: *L*. *donovani*, *L*. *panamensis*, *L*. *braziliensis*, *L*. *major* and *L*. *infantum*. The NOS-like alignment showed high identity among these species, confirming a biological role in the maintenance of parasite (S1 Fig). Additionally, a BLAST search for NOS-like (LmxM.19.1450) in the TriTryp database showed an identity of 99% with a contig of *L*. *(L*.*) amazonensis* MHOM/BR/71973/M2269 (KE391486.1). And a low identity was detected with *Leptomonas seymouri*, *T*. *cruzi* genome database (Dm28c), *T*. *vivax* (Y486), 74.2%, 21% and 16.2%, respectively (TriTryp database).

**Fig 2:**
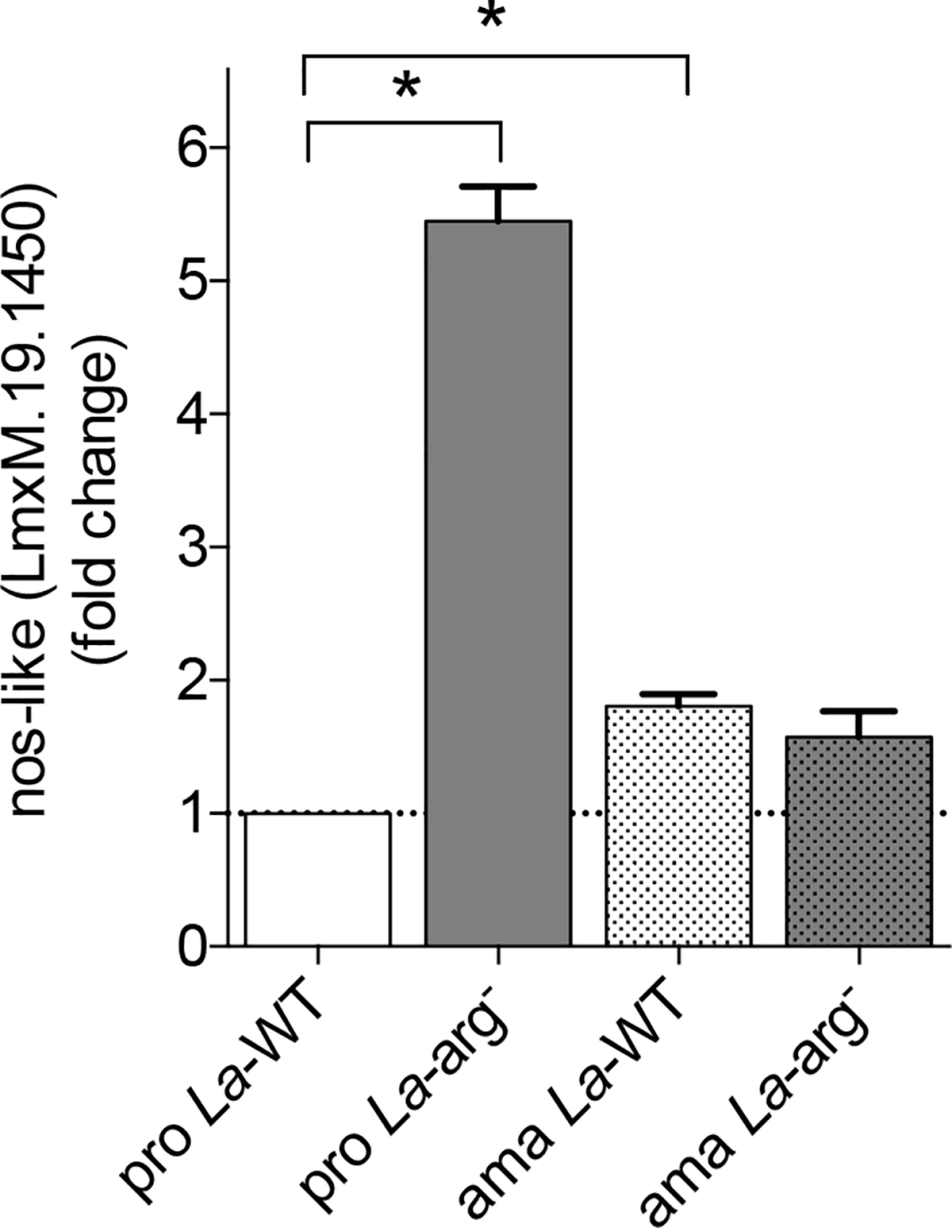
Nos-like mRNA expression by RT-qPCR. The *nos-like* mRNA levels were based on quantification of the target and were normalized by *gapdh* expression in promastigotes (pro) and amastigotes (ama) of *La*-WT and *La*-arg^-^. The values are the mean ± SEM of three independent biological replicates (n4-6). (*) p < 0.05.

In addition, we analyzed the expression of *nos-like* by RT-qPCR in *La*-WT and *La*-arg^-^ promastigotes and axenic amastigotes (Fig 2). As expected, an increase amount of *nos-like* was detected in *La*-arg^-^ promastigotes when compared to *La*-WT.

Then, we analyzed the gene differential expression of other oxidoreductases identified through RNA-seq analysis. In Table 1, quinone oxidoreductase (LmxM.03.0570/EC 1.6.5.5) was down-regulated in *La*-arg^-^ promastigotes compared to *La*-WT promastigotes. No differential expression gene was observed among the other oxidoreductases (Table 1).

In *La*-arg^-^ axenic amastigotes compared to *La*-WT axenic amastigotes showed an up-regulation of FAD dependent oxidoreductase (LmxM.36.5660), and modest up-regulation of NADPH:ubiquinone oxidoreductase (Lmx>05.0980/EC 1.6.5.3), NADPH:ubiquinone oxidoreductases (LmxM.18.1480/EC 1.6.5.3, LmxM.20.0120/EC 1.6.5.3), NADH:adrenodoxin oxidoreductases (LmxM.21.0420/EC 1.18.1.6). No differential expression gene was observed among the other oxidoreductases (Table 2).

In *La*-WT axenic amastigotes compared to promastigotes presented an up-regulation of NADH:flavin oxidoreductase/NADH oxidase (LmxM.12.1130/EC 1.3.1.42), NADH:adrenodoxin oxidoreductase (LmxM.31.2210) and oxidoreductases-like (LmxM.32.1770 and Lmx.36.4170/EC 1.6.5.5). No differential expression gene was observed among the other oxidoreductases (Table 3).

Finally, in *La*-arg^-^ axenic amastigotes compared to promastigotes identified an up-regulation of quinone oxidoreductases (LmxM.03.0570/EC 1.6.5.5), NADH:flavin oxidoreductase/NADH oxidase (LmxM.12.1130/EC 1.3.1.42) as well as oxidoreductases-like (LmxM.23.1590, LmxM.28.0949, LmxM.32.1770, LmxM.36.3230/EC 1.1.99.1, LmxM.36.4170/EC 1.6.5.5), NADH:adrenodoxin oxidoreductases (LmxM.21.0420/EC 1.18.1.6, Lmx.31.2210 and LmxM.33.4320) and FAD-dependent oxidoreductase (LmxM.36.5660) (Table 4).

### The increase in NO production is arginase-dependent and indicative of metacyclogenesis in *L*. *(L*.*) amazonensis* promastigotes

**Fig 3:**
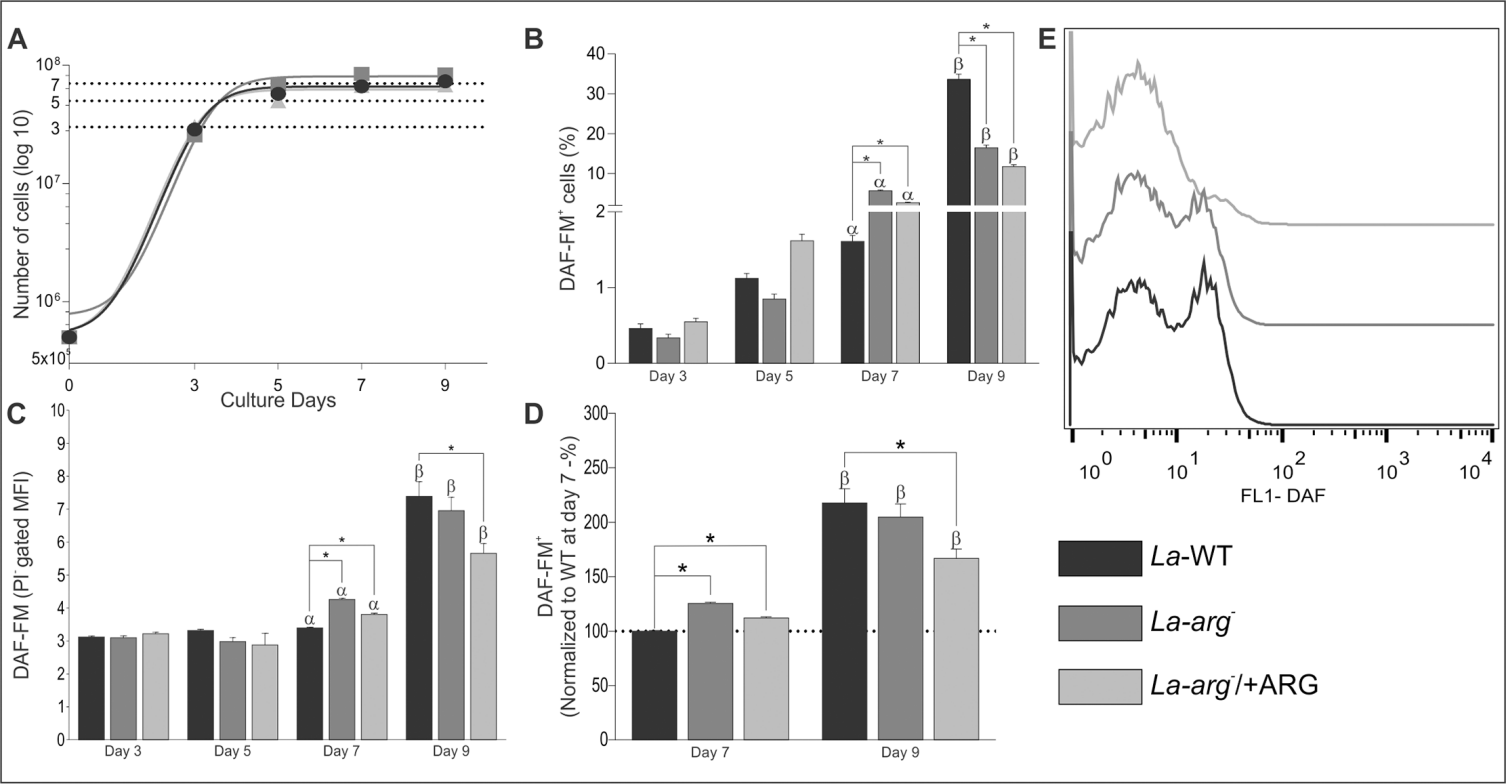
Growth curve and NO production in *La*-WT, *La*-arg^-^ and *La*-arg^-^/+ARG on days 3, 5, 7 and 9. *L*. *(L*.*) amazonensis* promastigotes wild-type (*La-*WT—black); arginase null knockout (*La-arg*^-—^dark gray) and the arginase addback (*La-arg*^-^/+ARG–light gray) growth curve and the NO production by DAF-FM labeling in flow cytometry. A total of 2x10^6^ cells was labeled with 1 ng/mL propidium iodide (PI) and was analyzed using FACSCalibur (Becton Dickinson). The viable cells (PI^-^) were used for the analysis of the frequency of DAF-FM-labeled cells and the mean fluorescence (MFI). **(A)** Growth curves and the establishment of the logarithmic growth phase (day 3); early-stationary (day 5), mid-stationary (day 7) and late-stationary phase (day 9). **(B)** Frequency of DAF-FM^+^ cells. Each bar represents the mean of the percent ±SEM of DAF-FM^+^ cells (n = 3–9). **(C)** MFI of DAF-FM^+^ cells. Each bar represents the mean of the fluorescence intensity ±SEM of viable cells (n = 3–9). **(D)** Normalization of MFI values in relation to day 7 of *La-*WT (100%). **(E)** Histogram of MFI of the PI^-^ gate at the 9^th^ day of culture. These data were analyzed by Student’s *t*-test, and (*) p < 0.05, (α) p < 0.05 compared the day 7 with the days 3 and 5, (β) p < 0.05 compared the day 9 with the days 3, 5 and 7. The data are representative of 3 independent experiments.

To correlate the expression of *nos-like* transcript and enzyme activity, we quantified NO amount in *La*-WT, *La-arg*^*-*^, and *La-arg*^*-*^/+ARG, along days 3, 5, 7 and 9 of a promastigote growth curve. We observed that all parasites lines reached the mid-logarithmic growth phase on day 3, the early-stationary growth phase occurred on day 5, the mid-stationary on day 7 and the late-stationary growth phase on day 9 (Fig 3A).

We also analyzed the viability of cells by PI labeling using gating of unlabeled cells (PI^-^) (gate 1, S2 Fig); from this gating, we analyzed the DAF-FM^+^ cells (Fig 3B and gate 2, S2 Fig) and the mean of fluorescence intensity of DAF-FM using flow cytometry (Fig 3C and 3D, S2 Fig). Viable cells (PI^-^ cells) represented approximately 99% of the total population (S2 Fig) for all tested promastigotes; only viable cells were used for DAF-FM analysis.

Then, we evaluated the NO production by DAF-FM labeling at every counting day in *La-*WT promastigotes in mid-logarithmic and early-stationary growth phase (days 3 and 5). We did not observe NO production in early growth phase of the parasite (Fig 3B). The detectable NO production was observed on day 7 at low levels, and high levels were detected on day 9 (Fig 3B), indicating that NO production can be related to the growth phase of the parasite.

The frequency of DAF-FM+ cells was nearly 1% on days 3, 5 and 7 in promastigotes *La*-WT and increased to 30% on day 9 in promastigotes late-stationary growth phase. In *La-arg*^*-*^ promastigotes, NO-producing cells were induced at the mid-stationary growth phase (day 7), showing nearly 5-fold increase compared to *La*-WT (Fig 3B). Similarly, the mean DAF-FM per cell was 1.3-fold increase in *La-arg*^*-*^ at the mid-stationary growth phase compared to *La*-WT (Fig 3C). In promastigotes *La*-WT at the late-stationary growth phase, the mean levels of DAF-FM per cell 2.5-fold increase compared to those on days 3, 5 or 7. The absence of arginase activity (*La-arg*^*-*^) or reduced arginase activity (*La*-arg^-^/+ARG) compared with *La*-WT [20] enabled an increase in DAF-FM^+^ cells in promastigotes at the mid-stationary growth phase, and the frequency was maintained in late-phase promastigotes, lower than *La*-WT, but the amount of NO per cell was increased in the absence of arginase activity (Fig 3B–3E). Altogether, these results suggested that NO production in *L*. *(L*.*) amazonensis* promastigotes is dependent on arginase activity and varies according to the growth phase of the parasite.

**Fig 4:**
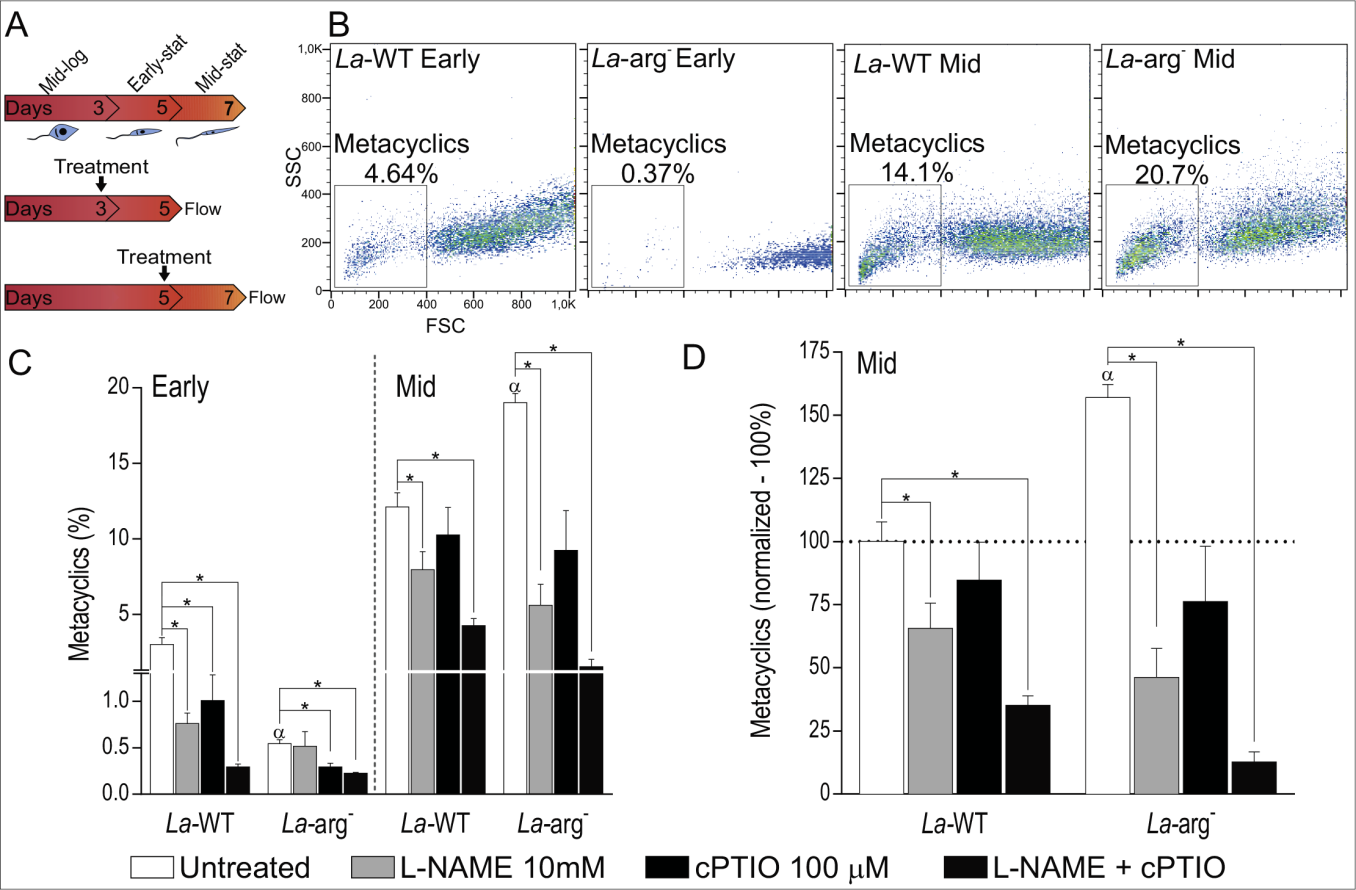
Inhibition of NOS activity or NO amount and metaclyclogenesis in *La*-WT and *La*-arg^-^ promastigote. (A) *L*. *(L*.*) amazonensis* wild-type (*La-*WT) and arginase null knockout (*La*-arg^-^) at logarithmic (log—day 3) and early-stat (early-stationary—day 5) phase promastigotes were untreated (white) or treated with 10 mM of L-NAME (light gray), 100 μM of cPTIO (dark gray) and L-NAME plus cPTIO (dark). After 2 days of culture metacyclic promastigotes forms were quantified by flow cytometry. (B) Dot plot of SSC and FSC features representative of metacyclics promastigote forms viable cells analyzed using FACSCalibur (Becton Dickinson). (C) Frequency of metacyclics promastigotes. Each bar represents the mean of the percent ±SEM of metaclyclic cells (n = 3–9). (D) Normalization of the percentage of metacyclic forms in relation to log phase of *La-*WT (100%). These data were analyzed by Student’s *t*-test, and (*) p < 0.05, (α) p < 0.05 compared the *La*-WT to *La*-arg^-^. The data are representative of 3 independent experiments (n = 6–9).

To analyze the function of NOS-like activity and NO production in metacyclogenesis, we used L-NAME (as NOS inhibitor), cPTIO (as NO scavenger) or L-NAME plus cPTIO treatments in mid-log (day 3) or early-stationary (day 5) growth phase of *La*-WT and *La-arg*^*-*^ promastigotes (Fig 4A).

The frequency of metacyclic forms 4-fold from increased in early- to mid -stationary promastigote growth phase in *La*-WT of (untreated, Fig 4B and 4C). The inhibition of NOS activity or reduction of NO amount during the differentiation of mid-log to early-stationary growth phase reduced the frequency of metacyclic forms around 74% (4-fold) and 67%(3-fold), respectively. NOS activity and NO reduced in 90% (10-fold) the frequency of metacyclic forms (Fig 4C and 4D). However, treatments with L-NAME, cPTIO or L-NAME/cPTI during differentiation of early- to mid-stationary growth phase reduced this metacyclic frequency to nearly 34% (1.5-fold), 17% (1.2-fold) and 65% (2.8-fold), respectively (Fig 4C and 4D).

The absence of arginase activity reduced the frequency of metacyclic forms in early-stationary phase, but increased in mid-stationary. Despite that, we observed a decrease in frequency of metacyclic forms when NOS activity and NO was reduced in *La-arg*^*-*^ (Fig 4C). The reduction on the frequency of metacyclics was pronounced during differentiation of early- to mid-stationary phase treated with L-NAME, cPTIO or/and L-NAME/cPTIO, to reduced 74%, 53% and 92%, respectively (Fig 4C and 4D). Thoroughly, these results correlated the NOS activity and NO levels to metacyclogenesis, as well as to dependence of arginase activity.

### The increase in NO production in *L*. *(L*.*) amazonensis* axenic amastigotes

**Fig 5:**
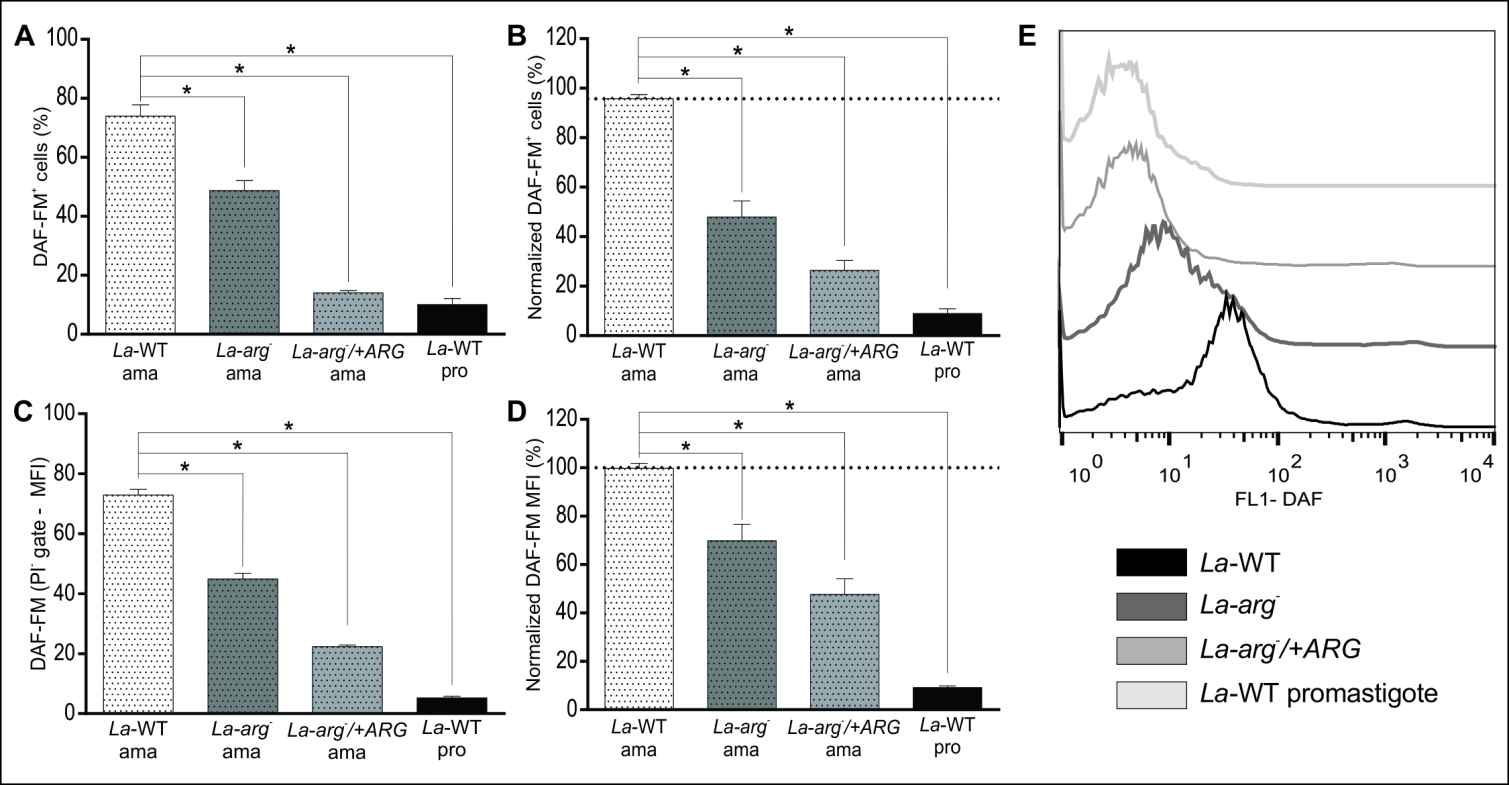
*L*. *(L*.*) amazonensis* axenic amastigotes produced NO in a higher amount than promastigotes. *La-*WT, *La-arg*^*-*^ and *La*-*arg*^*-*^/+ARG promastigotes at the early-stationary phase (day 5 – 5x10^6^ cells/10 mL) were differentiated into amastigotes by changing the M199 medium to pH 5.5 and maintaining the culture at 34°C during 4 days. Next, the cells were labeled with 1 μM DAF-FM and 1 ng/mL PI and were analyzed by flow cytometry. *La-*WT promastigotes in the late-stationary phase were used as a positive control. The viable cells (PI^-^) were used to analyze the frequency of DAF-FM^+^ cells and MFI. **(A)** Frequency of DAF-FM^+^ cells. These are representative of 1 independent experiment (n = 3–4). **(B)** Normalization of the percentage of DAF-FM^+^ *La-arg*^*-*^ and *La*-*arg*^*-*^/+ARG in relation to *La-*WT (100%). These are representative of 3 independent experiments (n = 9). **(C)** MFI of DAF-FM^+^ cells. These are representative of 1 independent experiment (n = 3–4). **(D)** Normalization of the MFI values of *La-arg*^*-*^ and *La*-*arg*^*-*^/+ARG in relation to *La-*WT (100%). These data are representative of 3 independent experiments (n = 9). These data were analyzed were analyzed by one-way ANOVA and Tukey’s comparison post-test. Significance *p-*value is represented by *, p<0.05. **(E)** Histogram of MFI of DAF-FM.

*La*-WT, *La-arg*^*-*^ and *La*-arg^-^/+ARG promastigotes in the early-stationary growth phase were subjected to amastigote differentiation in M 199 medium, at 34°C and pH 5.5, for 4 days. The differentiation to amastigotes forms was confirmed by amastins gene expression (LmxM.08.00760, LmxM.08.0800 and LmxM.33.0960) by RT-qPCR. The amastins transcripts were increased in axenic amastigotes than stationary-phase promastigotes of *La*-WT or *La-arg*^*-*^, with exception of LmxM.33.0960 in *La-arg*^*-*^ (S3 Fig). Next, the axenic amastigotes were labeled with DAF-FM and PI, as described for promastigotes. The viable cells (PI^-^) represented approximately 95% of the total population (S4 Fig). *La-*WT amastigotes represented the higher population labeled with DAF-FM, and approximately 75% of viable cells were DAF-FM^+^ (Fig 5A), a value higher than promastigotes. Compared to *La-*WT, NO-producing amastigotes were lower in *La*-arg^-^ (50%) and *La*-arg^-^/+ARG (70%) (Fig 5A and 5B). The *La*-WT promastigotes in the late-stationary phase were used as the positive control of this experiment.

Mean of fluorescence intensity (MFI) analysis of the tested amastigotes showed the same pattern regarding the percentage of cells producing NO. *La-*WT amastigotes was 2-fold and 3.5-fold increased amount of NO than *La-arg*^*-*^ and *La*-arg^-^/+ARG, respectively (Fig 5C–5E). The NO production in *La-*WT amastigotes was 10-fold increased than *La-*WT promastigotes. These results showed a higher production of NO in axenic amastigotes of *L*. *(L*.*) amazonensis* and the absence or reduction of arginase activity led to a reduction of NO production.

**Fig 6:**
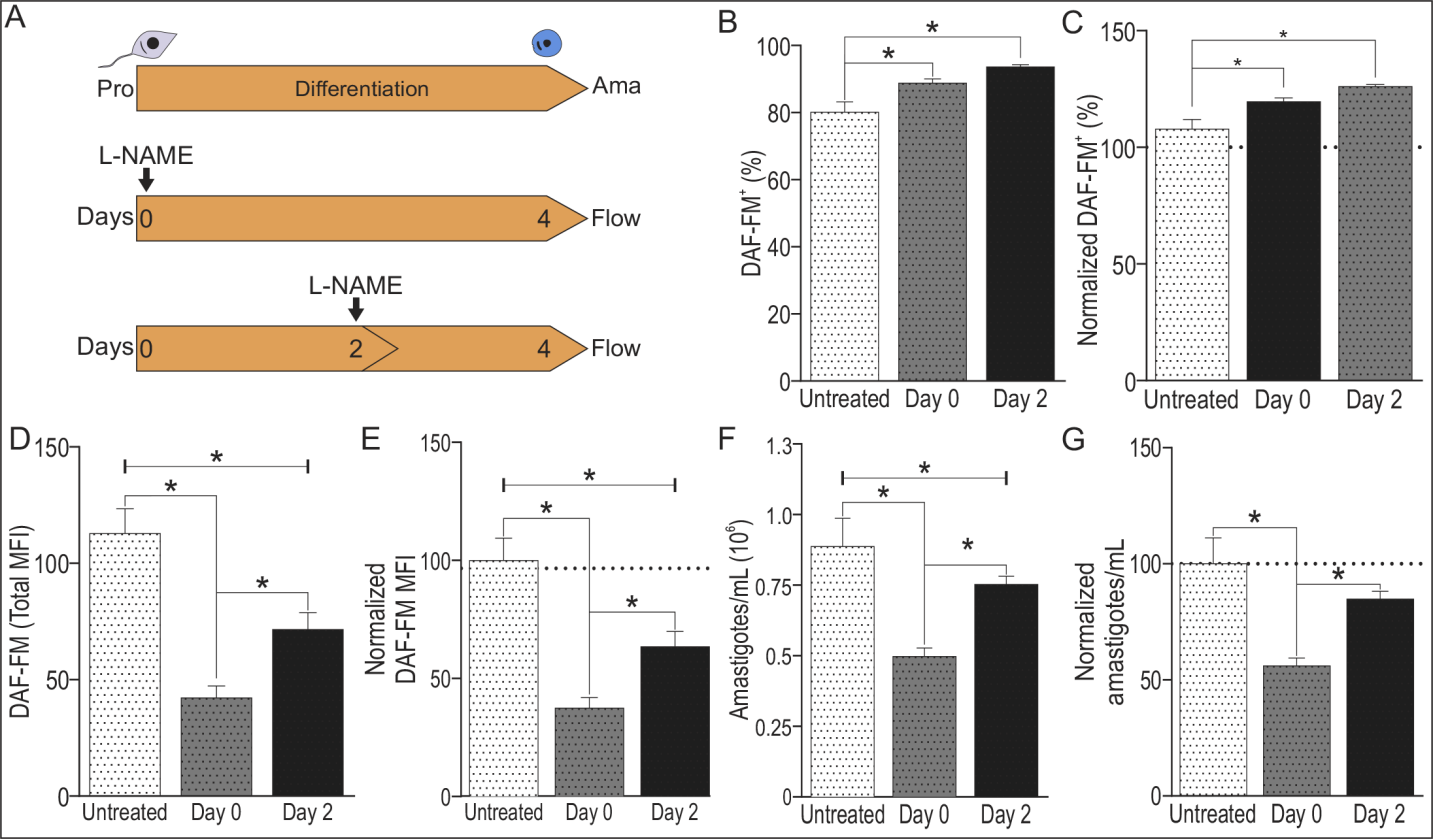
Inhibition of NOS activity during *La*-WT and *La*-arg^-^ amastigotes differentiation. (A) *L*. *(L*.*) amazonensis La-*WT at early-stationary phase promastigotes (pro) were differentiated into amastigotes (ama), by changing the M199 medium to pH 5.5 and maintaining the culture at 34°C during 4 days, untreated (white) or treated with 10 mM of L-NAME (light gray) at day 0 or day of culture. Next, a total of 2x10^6^ cells was labeled with 1 μM DAF-FM and 1 ng/mL propidium iodide (PI) and analyzed using FACSCalibur (Becton Dickinson). B) Frequency of of DAF-FM^+^ cells. (C) Normalization of the percentage of DAF-FM^+^ cells in relation to *La-*WT-untreated (100%). (D) MFI of DAF-FM^+^ cells. (E) Normalization of the MFI of DAF-FM^+^ cells in relation *La-*WT-untreated (100%). (F) Number of amastigotes forms. (G) Normalization of number of amastigote forms in relation *La-*WT-untreated (100%). Each bar represents the mean of the values ±SEM (n = 3–9). These data were analyzed by Student’s *t*-test, and (*) p < 0.05. The data are representative of 3 independent experiments.

The role of NOS activity and NO production during *La-*WT amastigotes differentiation was evaluated using a L-NAME (as NOS inhibitor) at day 0 or day 2 of culture of early-stationary promastigotes subjected to amastigotes differentiation for 4 days, at 34°C and pH 5.5 (Fig 5A). As observed previously, viable cells represented nearly 80% of total population of untreated or L-NAME treated cells. The frequency of DAF-FM^+^ cells presented a 10% slight increase after L-NAME treatment, (Fig 6B and 6C). However, the inhibition of NOS activity by L-NAME reduced the amount of NO per cell, in 58% and 29% in cells treated at day 0 or day 2, respectively (Fig 6C and 6D). The reduction in amastigotes numbers was pronounced after L-NAME treatment at day 0 compared to day 2 (Fig 6E and 6F), correlating reduction in NOS activity and NO production to number of amastigotes. These data revealed the NO production in the initial steps of amastigotes differentiation correlating with the replication of amastigotes forms.

## Discussion

The biological function of NO in trypanosomatids is still not completely understood. The main controversy is to understand why the parasite produces NO, the same molecule produced by the host macrophages to kill them [19,22,44]. The key to answer this question can be related to the NO concentration. NO can perform multiple actions depending on its concentration, production time and exposure time. Previous studies with *T*. *cruzi* demonstrated that low NO concentration (nM levels) resulted in the induction of cGMP production and post-translational modifications, interfering in several biological processes, such as PKC signaling, cytochrome C regulation, caspase-cascade inactivation, protein degradation and control of the redox environment [45–50]. On the other hand, at high concentrations (mM), NO can be toxic, passing across various chemical reactions [26,51].

Physiological processes mediated by NO are modulated due the characteristics of the free radical with a short half-life [52] and quick diffusion away from the site of its synthesis [27]. Other modulation factor of these processes includes the source of L-arginine, presence of molecular oxygen and cofactors, such as NADPH, FAD, heme, FMN and BH4, the presence an active NOS, and the intracellular or extracellular environment limits/boundaries for the NO action [26,53,54]. Additionally, NO can react with other free radical species, such as superoxide, thiol radicals or lipid peroxides, or metal-coupled proteins such as hemoglobin [51,53].

In this work, we focused on oxidoreductases expression. Oxidoreductase is a class of enzymes that catalyze biological oxidation/reduction reactions [55]. The transcriptomic profiling analysis identified several oxidoreductases: (1) NADH:quinone oxidoreductase, previous described with important role in yeasts and prokaryotes to maintain the [NADH]/[NAD+] balance and for the entrance point of electrons into the respiratory chains of complex I coupling to the pumping of protons across the inner mitochondrial membrane [56,57]; (2) NADPH:ubiquinone oxidoreductases, previously described as related to the transfer of electrons from NADH to ubiquinone with the translocation of protons across the membrane in the respiratory complex I [58]; (3) electron transfer oxidoreductase or electron-transfer flavoprotein:ubiquinone oxidoreductase, previously described as a component of the mitochondrial respiratory chain, which catalyzes the electron-transfer of flavoprotein to the ubiquinone pool, as the electron acceptor [59]; (4) NADH:adrenodoxin oxidoreductases, previously described to initiate electron transport for cytochrome P450 receiving electrons from NADPH to reduce O2 to a superoxide radical [60]; (5) NADH:flavin oxidoreductase/NADH oxidase, previously described catalyzing the reduction of free flavins by NADPH or NADH; such enzymes are present in all microorganisms [61,62]; and (6) FAD-dependent oxidoreductase previously described as one of the assembly factors in the respiratory chains of complex I [63].

We highlighted one of them, the oxidoreductase-like (LmxM.19.1450/ EC 1.14.13.39). This oxidoreductase-like appeared up-regulated in *La-arg*^-^ promastigotes compared to *La*-WT promastigotes and in *La*-WT axenic amastigotes compared to *La*-WT promastigotes, indicating a modulation during promastigote to amastigote differentiation as well as a dependence on arginase activity. According to the conserved domains content and to the metabolome data pointing to the expression of an enzyme (EC 1.14.13.39), which could be involved in the production of NO and citrulline [18], we suggested this oxidoreductase as a NOS-like. In addition, the multiple alignment revealed this NOS-like conserved among *Leishmania* spp.

**Fig 7:**
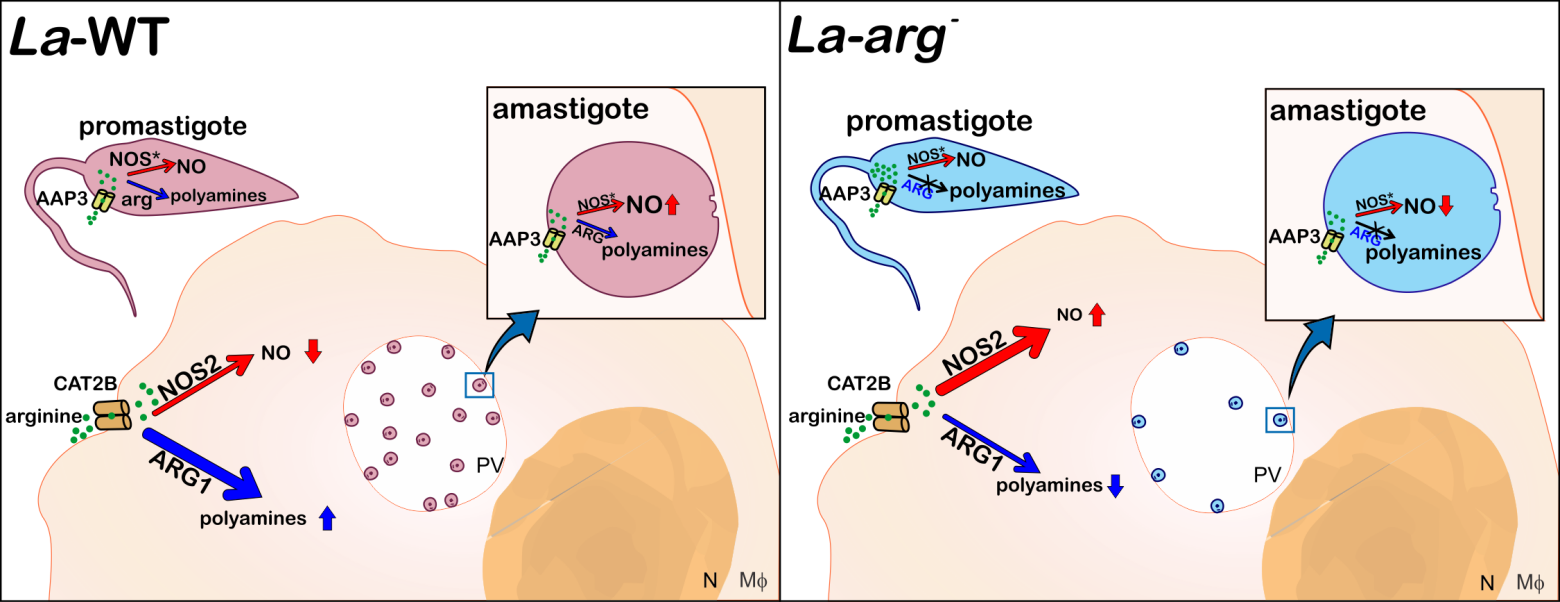
Schematic representation of *L*. *(L*.*) amazonensis* promastigote and amastigote NO production in the infection context. In *L*. *(L*.*) amazonensis*, the AAP3 transporter takes up L-arginine, and this amino acid is metabolized by arginase into ornithine to produce polyamines or via NOS-like to produce NO. *La*-WT-infected macrophages increase the activity of the polyamine pathway, increasing the expression of the L-arginine transporter from the host (CAT2B) and parasite (AAP3), arginase1 (host) and parasite-arginase to metabolizing L-arginine to produce polyamines, consequently sustaining the basal level of NOS2 expression and NO production. Indeed, the *La*-WT amastigote differentiation induces NOS-like expression and NO production, higher levels than promastigotes, but lower than those produced by macrophages. The lack of arginase activity in promastigotes increases the L-arginine levels, even that the reduction in AAP3 expression, inducing the expression of NOS-like, NO and citrulline production. The *La*-arg^—^infected macrophages present lower levels of CAT2B and AAP3, which could reduce L-arginine uptake, and lower the levels of arginase 1 and polyamine production compared with *La*-WT infection allowing the increase in NOS2 expression and NO production, reducing infectivity. In addition, the *La*-arg^-^ amastigote differentiation induces NOS-like expression but not AAP3 expression and L-arginine uptake sufficient to increase NO production compared with *La*-WT promastigotes. PV: parasitophorous vacuole.

The fate of *Leishmania* infection in the mammalian host depends on dual role of macrophage L-arginine metabolism: the production of NO promoting the killing of the pathogen [19,22,44], or the production of ornithine, the polyamine precursor allowing the parasite replication [19,44,64]. *Leishmania* presents its own machinery to metabolize L-arginine: the AAP3 for the uptake and the arginase activity to metabolize the amino acid to polyamines production, which are essential for *in vitro* proliferation, as shown for *L*. *(L*.*) amazonensis* [6,8,20], *L*. *donovani* [65], *L*. *(L*.*) mexicana* [66] and *L*. *(L*.*) major* [66,67]. The lack of arginase activity reduces AAP3 and arginase levels and the infectivity of *L*. *(L*.*) amazonensis* [20,22], *L*. *donovani* [65], *L*. *(L*.*) mexicana* [66] and *L*. *(L*.*) major* [66,67]. In infected macrophages, L-arginine can be metabolized by both arginases, from the *Leishmania* (*La-*ARG) and from the macrophage (ARG1), supplying the polyamine pathway and enabling parasite survival [7,8,19,22,66]. Depending on substrate competition, L-arginine can be hydrolyzed by the macrophage NOS2 or by the parasite NOS-like [13–15,17,18]. Based on that, we proposed a schematic representation on the fate of the infection (Fig 7).

The attenuation of infectivity in *La-arg*^-^ [20,22], as well as in *L*. *mexicana-arg*^*-*^ [68] was associated with NO overexpression by host macrophages but not in *L*. *major-arg*^*-*^ [69]. The depletion of L-arginine in macrophages can inhibit both polyamines and NO production. In addition, the lower infectivity of *La-arg*^*-*^ could correlate with lower levels of CAT2B [21] or AAP3 expression and amino acid uptake [70], reducing L-arginine availability for ARG1 in infected macrophages, reducing parasite survival. In this way, infectivity could be recovered by putrescine supplementation [21].

Our data showed NO production in *L*. *(L*.*) amazonensis* stationary phase promastigotes corroborating previous studies that detected NO in promastigotes of *L*. *(L*.*) amazonensis* M2269, *L*. *(V*.*) braziliensis* M2903, *L*. *(L*.*) chagasi* PP75, *L*. *(L*.*) donovani* AG83, *L*. *(L*.*) donovani* DD8, *L*. *(L*.*) infantum* IPT1, *L*. *(L*.*) major* 5ASKH, *L*. *(L*.*) mexicana* MIC and *L*. *(V*.*) panamensis* LS94 [17]. Indeed, the suggestion of NOS-like expression in *Leishmania* corroborates other studies that *Leishmania* harbors NOS-like activity to produce NO [13–15,17]. Genestra et al. (2006) purified a protein that exhibited NOS activity in *L*. *(L*.*) amazonensis* and showed similarities to *T*. *cruzi* NOS and neuronal NOS (nNOS). In addition, this group demonstrated NOS-like activity as Ca^2+^ and calmodulin dependent, corresponding to a constitutive form of protein [15]. Leon’s group also showed an increased amount of NO in *L*. *(L*.*) amazonensis* metacyclic form and in axenic amastigotes [15]. Our data correlated the NO production signaling to metacyclogenesis, since reduction in NOS activity and NO levels reduced the metacyclic differentiation, as well as reduced the amastigotes differentiation and growth.

The NO could confer parasite protection altering the steady state of promastigotes and amastigotes to resist to toxicity signals mediate by macrophage inflammatory. Corroborating with this idea, some studies show the influence of NO in protecting parasites from antimony-mediated oxidative stress promoting a higher H_2_O_2_ scavenging activity and increase in thiol levels [71]. Indeed, NO induction induces heat shock proteins expression [72] and could inhibit molecules involved in the apoptosis signal, such as cysteine proteinases [73], cis-aconitase and glyceraldehydes-3-phosphate dehydrogenase [74,75]. Although, NO-resistant parasites are able to differentiate from promastigotes to amastigotes and from amastigotes to promastigotes, but the differentiation of NO-resistant promastigotes to amastigotes occurs in lower levels than non-resistant parasites, probably by increased levels of 6-phosphogluconate dehydrogenase involved in NADPH and ribose-5-phosphate generation resulting in rapid consumption of glucose that accelerates the entry in stationary phase of growth [74].

Meanwhile, the high levels of NO detected in late-stationary growth phase of *L*. *(L*.*) amazonensis* promastigotes support the link between NO production and metacyclogenesis, similar to the previous observation that correlated a high amount of NO production in culture with a high number of metacyclic forms as well as with the infectivity [13]. In addition, L-arginine starvation of promastigotes *L*. *(L*.*) amazonensis* at the logarithmic growth phase reduced ornithine and putrescine levels [18]. By contrast, the absence of arginase activity increased the L-arginine availability and the citrulline levels, but decreased the levels of ornithine and putrescine in promastigotes [6,18,20]. As a consequence, it promoted NO production in the earlier steps of the stationary phase growth phase of promastigote differentiation (this communication). This evidence points to the importance of the competition for the same substrate, L-arginine, by arginase and NOS-like during metacyclogenesis and amastigotes differentiation, influencing the NO production.

## Supporting information

S1 Fig Alignment of the nitric oxide synthase-like (LmxM.19.1450) from *L*. *mexicana* and their orthologs. Multiple alignment of the amino acid sequence of the nitric oxide synthase-like NOS-like of. *L*. *amazonensis* (Lam), *L*. *mexicana* (Lmx), *L*. *donovani* (Ldo), *L*. *panamensis* (Lpa), *L*. *braziliensis* (Lbr), *L*. *major* (Lmj) and *L*. *infantum*. The identical amino acids are highlighted in gray. The alignment was performed based on NCBI multiple alignment tool.
(TIF)

S2 Fig Experimental procedures for DAF-FM analysis in promastigotes by flow cytometry. The DAF-FM labeling of *La*-WT, *La*-arg^-^ and *La*-arg^-^/+ARG promastigotes and analysis were performed as follows: (1) DAF-FM labeling followed by PI (propidium iodide) staining; (2) The data were acquired using a FACSCalibur (BD) flow cytometer followed by analysis of the resulting pattern of labeling: PI^-^DAF^-^, PI^-^DAF^+^, PI^+^DAF^-^ and PI^+^DAF^+^. Using FlowJo software (as described on methods section), the cells were gated based on the Forward Scatter (FSC) and the PI label was used to gate viable cells (PI^-^); (3) Using viable cells, DAF^+^ populations were selected for frequency analysis, and (4) the population producing NO was gated based on the histogram of the Mean of Fluorescence Intensity (MFI).
(TIF)

S3 Fig Experimental validation of amastigotes differentiation. (A) The *amastins* LmxM.08.00760 (A), LmxM.0800800 (B) and LmxM.33.0960 (C) mRNA levels were based on quantification of the target and were normalized by *gapdh* expression in promastigotes (pro) and amastigotes (ama) of *La-*WT and *La*-arg^-^. The values are the mean ± SEM of three independent biological replicates (n = 4–6). (*) p < 0.05
(TIF)

S4 Fig Experimental procedures for DAF-FM analysis from amastigotes by flow cytometry. DAF-FM labeling of *La*-WT, *La*-arg- and *La*-arg^-^/+ARG amastigotes and analysis were performed as follows: (1) DAF-FM labelling followed by PI (propidium iodide) staining; (2) The data were acquired on a FACSCalibur (BD) flow cytometer with analysis of the resulting pattern of labelling: PI^-^DAF^-^, PI^-^DAF^+^, PI^+^DAF^-^ and PI^+^DAF^+^. Using FloJo software (as described on methods section), the cells were gated based on the Forward Scatter (FSC) and PI labeling was used to gate viable cells (PI^-^); (3) Using viable cells, DAF^+^ populations were selected for frequency analysis, and (4) the population producing NO was gated based on the histogram of the Mean Fluorescence Intensity (MFI).
(TIF)

## References

[1] Bogdan C (2008) Mechanisms and consequences of persistence of intracellular pathogens: leishmaniasis as an example. Cell Microbiol 10: 1221–1234. doi: 10.1111/j.1462-5822.2008.01146.x 18363880 10.1111/j.1462-5822.2008.01146.x

[2] Liese J, Schleicher U, Bogdan C (2008) The innate immune response against Leishmania parasites. Immunobiology 213: 377–387. doi: 10.1016/j.imbio.2007.12.005 18406382 10.1016/j.imbio.2007.12.005

[3] Sacks D, Kamhawi S (2001) Molecular aspects of parasite-vector and vector-host interactions in leishmaniasis. Annu Rev Microbiol 55: 453–483. doi: 10.1146/annurev.micro.55.1.453 11544364 10.1146/annurev.micro.55.1.453

[4] Teixeira DE, Benchimol M, Rodrigues JC, Crepaldi PH, Pimenta PF, de Souza W (2013) The cell biology of Leishmania: how to teach using animations. PLoS Pathog 9: e1003594 doi: 10.1371/journal.ppat.1003594 24130476 10.1371/journal.ppat.1003594 PMC3795027

[5] Séguin O, Descoteaux A (2016) Leishmania, the phagosome, and host responses: The journey of a parasite. Cell Immunol 309: 1–6. doi: 10.1016/j.cellimm.2016.08.004 27531526 10.1016/j.cellimm.2016.08.004

[6] Castilho-Martins EA, Laranjeira da Silva MF, dos Santos MG, Muxel SM, Floeter-Winter LM (2011) Axenic Leishmania amazonensis promastigotes sense both the external and internal arginine pool distinctly regulating the two transporter-coding genes. PLoS One 6: e27818 doi: 10.1371/journal.pone.0027818 22114701 10.1371/journal.pone.0027818 PMC3218042

[7] da Silva MF, Zampieri RA, Muxel SM, Beverley SM, Floeter-Winter LM (2012) Leishmania amazonensis arginase compartmentalization in the glycosome is important for parasite infectivity. PLoS One 7: e34022 doi: 10.1371/journal.pone.0034022 22479507 10.1371/journal.pone.0034022 PMC3316525

[8] da Silva ER, Castilho TM, Pioker FC, Tomich de Paula Silva CH, Floeter-Winter LM (2002) Genomic organisation and transcription characterisation of the gene encoding Leishmania (Leishmania) amazonensis arginase and its protein structure prediction. Int J Parasitol 32: 727–737. 12062491 10.1016/s0020-7519(02)00002-4

[9] Green SJ, Crawford RM, Hockmeyer JT, Meltzer MS, Nacy CA (1990) Leishmania major amastigotes initiate the L-arginine-dependent killing mechanism in IFN-gamma-stimulated macrophages by induction of tumor necrosis factor-alpha. J Immunol 145: 4290–4297. 2124240

[10] Green SJ, Meltzer MS, Hibbs JB Jr., Nacy CA (1990) Activated macrophages destroy intracellular Leishmania major amastigotes by an L-arginine-dependent killing mechanism. J Immunol 144: 278–283. 2104889

[11] Liew FY, Li Y, Moss D, Parkinson C, Rogers MV, Moncada S (1991) Resistance to Leishmania major infection correlates with the induction of nitric oxide synthase in murine macrophages. Eur J Immunol 21: 3009–3014. doi: 10.1002/eji.1830211216 1721024 10.1002/eji.1830211216

[12] Goldman-Pinkovich A, Balno C, Strasser R, Zeituni-Molad M, Bendelak K, Rentsch D, et al (2016) An Arginine Deprivation Response Pathway Is Induced in Leishmania during Macrophage Invasion. PLoS Pathog 12: e1005494 doi: 10.1371/journal.ppat.1005494 27043018 10.1371/journal.ppat.1005494 PMC4846328

[13] Genestra M, de Souza WJ, Cysne-Finkelstein L, Leon LL (2003) Comparative analysis of the nitric oxide production by Leishmania sp. Med Microbiol Immunol 192: 217–223. doi: 10.1007/s00430-003-0176-z 12827512 10.1007/s00430-003-0176-z

[14] Genestra M, Guedes-Silva D, Souza WJ, Cysne-Finkelstein L, Soares-Bezerra RJ, Monteiro FP, et al (2006) Nitric oxide synthase (NOS) characterization in Leishmania amazonensis axenic amastigotes. Arch Med Res 37: 328–333. doi: 10.1016/j.arcmed.2005.07.011 16513480 10.1016/j.arcmed.2005.07.011

[15] Genestra M, Souza WJ, Guedes-Silva D, Machado GM, Cysne-Finkelstein L, Bezerra RJ, et al (2006) Nitric oxide biosynthesis by Leishmania amazonensis promastigotes containing a high percentage of metacyclic forms. Arch Microbiol 185: 348–354. doi: 10.1007/s00203-006-0105-9 16575586 10.1007/s00203-006-0105-9

[16] Sarkar A, Mandal G, Singh N, Sundar S, Chatterjee M (2009) Flow cytometric determination of intracellular non-protein thiols in Leishmania promastigotes using 5-chloromethyl fluorescein diacetate. Exp Parasitol 122: 299–305. doi: 10.1016/j.exppara.2009.04.012 19393240 10.1016/j.exppara.2009.04.012

[17] Sarkar A, Saha P, Mandal G, Mukhopadhyay D, Roy S, Singh SK, et al (2011) Monitoring of intracellular nitric oxide in leishmaniasis: its applicability in patients with visceral leishmaniasis. Cytometry A 79: 35–45. doi: 10.1002/cyto.a.21001 21182181 10.1002/cyto.a.21001

[18] Castilho-Martins EA, Canuto GA, Muxel SM, da Silva MF, Floeter-Winter LM, Del Aguila C, et al (2015) Capillary electrophoresis reveals polyamine metabolism modulation in Leishmania (Leishmania) amazonensis wild type and arginase knockout mutants under arginine starvation. Electrophoresis. 10.1002/elps.201500114 26202519

[19] Wanasen N, Soong L (2008) L-arginine metabolism and its impact on host immunity against Leishmania infection. Immunol Res 41: 15–25. doi: 10.1007/s12026-007-8012-y 18040886 10.1007/s12026-007-8012-y PMC2639710

[20] Laranjeira-Silva MF, Zampieri RA, Muxel SM, Beverley SM, Floeter-Winter LM (2012) Leishmania amazonensis arginase compartmentalization in the glycosome is important for parasite infectivity. PLoS One 7: e34022 doi: 10.1371/journal.pone.0034022 22479507 10.1371/journal.pone.0034022 PMC3316525

[21] Laranjeira-Silva MF, Zampieri RA, Muxel SM, Floeter-Winter LM, Markus RP (2015) Melatonin attenuates Leishmania (L.) amazonensis infection by modulating arginine metabolism. J Pineal Res. 10.1111/jpi.12279 26383232

[22] Muxel SM, Laranjeira-Silva MF, Zampieri RA, Floeter-Winter LM (2017) Leishmania (Leishmania) amazonensis induces macrophage miR-294 and miR-721 expression and modulates infection by targeting NOS2 and L-arginine metabolism. Sci Rep 7: 44141 doi: 10.1038/srep44141 28276497 10.1038/srep44141 PMC5343489

[23] Alderton WK, Cooper CE, Knowles RG (2001) Nitric oxide synthases: structure, function and inhibition. Biochem J 357: 593–615. 11463332 10.1042/0264-6021:3570593 PMC1221991

[24] MacMicking J, Xie QW, Nathan C (1997) Nitric oxide and macrophage function. Annu Rev Immunol 15: 323–350. doi: 10.1146/annurev.immunol.15.1.323 9143691 10.1146/annurev.immunol.15.1.323

[25] Stuehr DJ (1999) Mammalian nitric oxide synthases. Biochim Biophys Acta 1411: 217–230. 10320659 10.1016/s0005-2728(99)00016-x

[26] Pacher P, Beckman JS, Liaudet L (2007) Nitric oxide and peroxynitrite in health and disease. Physiol Rev 87: 315–424. doi: 10.1152/physrev.00029.2006 17237348 10.1152/physrev.00029.2006 PMC2248324

[27] Wink DA, Hines HB, Cheng RY, Switzer CH, Flores-Santana W, Vitek MP, et al (2011) Nitric oxide and redox mechanisms in the immune response. J Leukoc Biol 89: 873–891. doi: 10.1189/jlb.1010550 21233414 10.1189/jlb.1010550 PMC3100761

[28] Planchet E, Kaiser WM (2006) Nitric oxide production in plants: facts and fictions. Plant Signal Behav 1: 46–51. 19521475 10.4161/psb.1.2.2435 PMC2633878

[29] Paveto C, Pereira C, Espinosa J, Montagna AE, Farber M, Esteva M, et al (1995) The nitric oxide transduction pathway in Trypanosoma cruzi. J Biol Chem 270: 16576–16579. 7542649 10.1074/jbc.270.28.16576

[30] Piacenza L, Peluffo G, Radi R (2001) L-arginine-dependent suppression of apoptosis in Trypanosoma cruzi: contribution of the nitric oxide and polyamine pathways. Proc Natl Acad Sci U S A 98: 7301–7306. doi: 10.1073/pnas.121520398 11404465 10.1073/pnas.121520398 PMC34663

[31] Pereira M, Soares C, Canuto GA, Tavares MF, Colli W, Alves MJ (2015) Down regulation of NO signaling in Trypanosoma cruzi upon parasite-extracellular matrix interaction: changes in protein modification by nitrosylation and nitration. PLoS Negl Trop Dis 9: e0003683 doi: 10.1371/journal.pntd.0003683 25856423 10.1371/journal.pntd.0003683 PMC4391712

[32] Basu NK, Kole L, Ghosh A, Das PK (1997) Isolation of a nitric oxide synthase from the protozoan parasite, Leishmania donovani. FEMS Microbiol Lett 156: 43–47. 9368359 10.1111/j.1574-6968.1997.tb12703.x

[33] Temporal RM, Cysne-Finkelstein L, Echevarria A, Silva-Goncalves AJ, Leon LL, Genestra MS (2005) Amidine derivatives and Leishmania amazonensis: an evaluation of the effect of nitric oxide (NO) production on the parasite-macrophage interaction. J Enzyme Inhib Med Chem 20: 13–18. doi: 10.1080/14756360400015207 15895679 10.1080/14756360400015207

[34] Fiebig M, Kelly S, Gluenz E (2015) Comparative Life Cycle Transcriptomics Revises Leishmania mexicana Genome Annotation and Links a Chromosome Duplication with Parasitism of Vertebrates. PLoS Pathog 11: e1005186 doi: 10.1371/journal.ppat.1005186 26452044 10.1371/journal.ppat.1005186 PMC4599935

[35] Hodgkinson VH, Soong L, Duboise SM, McMahon-Pratt D (1996) Leishmania amazonensis: cultivation and characterization of axenic amastigote-like organisms. Exp Parasitol 83: 94–105. doi: 10.1006/expr.1996.0053 8654556 10.1006/expr.1996.0053

[36] Cysne-Finkelstein L, Temporal RM, Alves FA, Leon LL (1998) Leishmania amazonensis: long-term cultivation of axenic amastigotes is associated to metacyclogenesis of promastigotes. Exp Parasitol 89: 58–62. doi: 10.1006/expr.1998.4276 9603489 10.1006/expr.1998.4276

[37] Bolger AM, Lohse M, Usadel B (2014) Trimmomatic: a flexible trimmer for Illumina sequence data. Bioinformatics 30: 2114–2120. doi: 10.1093/bioinformatics/btu170 24695404 10.1093/bioinformatics/btu170 PMC4103590

[38] Van der Auwera GA, Carneiro MO, Hartl C, Poplin R, Del Angel G, Levy-Moonshine A, et al (2013) From FastQ data to high confidence variant calls: the Genome Analysis Toolkit best practices pipeline. Curr Protoc Bioinformatics 43: 11.10.11–33. 25431634 10.1002/0471250953.bi1110s43 PMC4243306

[39] Kim D, Pertea G, Trapnell C, Pimentel H, Kelley R, Salzberg SL (2013) TopHat2: accurate alignment of transcriptomes in the presence of insertions, deletions and gene fusions. Genome Biol 14: R36 doi: 10.1186/gb-2013-14-4-r36 23618408 10.1186/gb-2013-14-4-r36 PMC4053844

[40] Trapnell C, Pachter L, Salzberg SL (2009) TopHat: discovering splice junctions with RNA-Seq. Bioinformatics 25: 1105–1111. doi: 10.1093/bioinformatics/btp120 19289445 10.1093/bioinformatics/btp120 PMC2672628

[41] Trapnell C, Roberts A, Goff L, Pertea G, Kim D, Kelley DR, et al (2012) Differential gene and transcript expression analysis of RNA-seq experiments with TopHat and Cufflinks. Nat Protoc 7: 562–578. doi: 10.1038/nprot.2012.016 22383036 10.1038/nprot.2012.016 PMC3334321

[42] Mortazavi A, Williams BA, McCue K, Schaeffer L, Wold B (2008) Mapping and quantifying mammalian transcriptomes by RNA-Seq. Nat Methods 5: 621–628. doi: 10.1038/nmeth.1226 18516045 10.1038/nmeth.1226 PMC13303166

[43] Aoki JI, Muxel SM, Zampieri RA, Laranjeira da Silva MF, Muller KE, Nerland A, et al (2017) RNA-seq transcriptional profiling of Leishmania amazonensis reveals an arginase-dependent gene expression regulation. PLoS Negl Trop Dis 11. 10.1371/journal.pntd.0006026 PMC5678721 29077741

[44] Laranjeira-Silva MF, Floeter-Winter LM (2014) Arginase in *Leishmania* In: Andre LS Santos MHB, d'Avila-Levy Claudia M, Kneipp Lucimar F, Sodre Catia L, editor. Proteins and Proteomics of Leishmania and Trypanossoma. Springer: Springer. pp. 103–118.

[45] Abello N, Kerstjens HA, Postma DS, Bischoff R (2009) Protein tyrosine nitration: selectivity, physicochemical and biological consequences, denitration, and proteomics methods for the identification of tyrosine-nitrated proteins. J Proteome Res 8: 3222–3238. doi: 10.1021/pr900039c 19415921 10.1021/pr900039c

[46] Hess DT, Stamler JS (2012) Regulation by S-nitrosylation of protein post-translational modification. J Biol Chem 287: 4411–4418. doi: 10.1074/jbc.R111.285742 22147701 10.1074/jbc.R111.285742 PMC3281651

[47] Nakagawa H, Komai N, Takusagawa M, Miura Y, Toda T, Miyata N, et al (2007) Nitration of specific tyrosine residues of cytochrome C is associated with caspase-cascade inactivation. Biol Pharm Bull 30: 15–20. 17202652 10.1248/bpb.30.15

[48] Souza JM, Choi I, Chen Q, Weisse M, Daikhin E, Yudkoff M, et al (2000) Proteolytic degradation of tyrosine nitrated proteins. Arch Biochem Biophys 380: 360–366. doi: 10.1006/abbi.2000.1940 10933892 10.1006/abbi.2000.1940

[49] Balafanova Z, Bolli R, Zhang J, Zheng Y, Pass JM, Bhatnagar A, et al (2002) Nitric oxide (NO) induces nitration of protein kinase Cepsilon (PKCepsilon), facilitating PKCepsilon translocation via enhanced PKCepsilon -RACK2 interactions: a novel mechanism of no-triggered activation of PKCepsilon. J Biol Chem 277: 15021–15027. doi: 10.1074/jbc.M112451200 11839754 10.1074/jbc.M112451200

[50] Ji Y, Neverova I, Van Eyk JE, Bennett BM (2006) Nitration of tyrosine 92 mediates the activation of rat microsomal glutathione s-transferase by peroxynitrite. J Biol Chem 281: 1986–1991. doi: 10.1074/jbc.M509480200 16314419 10.1074/jbc.M509480200

[51] Wink DA, Mitchell JB (1998) Chemical biology of nitric oxide: Insights into regulatory, cytotoxic, and cytoprotective mechanisms of nitric oxide. Free Radic Biol Med 25: 434–456. 9741580 10.1016/s0891-5849(98)00092-6

[52] Lowenstein CJ, Dinerman JL, Snyder SH (1994) Nitric oxide: a physiologic messenger. Ann Intern Med 120: 227–237. 8273987 10.7326/0003-4819-120-3-199402010-00009

[53] Hall CN, Garthwaite J (2009) What is the real physiological NO concentration in vivo? Nitric Oxide 21: 92–103. doi: 10.1016/j.niox.2009.07.002 19602444 10.1016/j.niox.2009.07.002 PMC2779337

[54] Lancaster JR Jr. (1997) A tutorial on the diffusibility and reactivity of free nitric oxide. Nitric Oxide 1: 18–30. doi: 10.1006/niox.1996.0112 9701041 10.1006/niox.1996.0112

[55] May SW (1999) Applications of oxidoreductases. Curr Opin Biotechnol 10: 370–375. doi: 10.1016/S0958-1669(99)80067-6 10449319 10.1016/S0958-1669(99)80067-6

[56] Melo AM, Bandeiras TM, Teixeira M (2004) New insights into type II NAD(P)H:quinone oxidoreductases. Microbiol Mol Biol Rev 68: 603–616. doi: 10.1128/MMBR.68.4.603-616.2004 15590775 10.1128/MMBR.68.4.603-616.2004 PMC539002

[57] Brandt U (2006) Energy converting NADH:quinone oxidoreductase (complex I). Annu Rev Biochem 75: 69–92. doi: 10.1146/annurev.biochem.75.103004.142539 16756485 10.1146/annurev.biochem.75.103004.142539

[58] Morina K, Schulte M, Hubrich F, Dorner K, Steimle S, Stolpe S, et al (2011) Engineering the respiratory complex I to energy-converting NADPH:ubiquinone oxidoreductase. J Biol Chem 286: 34627–34634. doi: 10.1074/jbc.M111.274571 21832062 10.1074/jbc.M111.274571 PMC3186356

[59] Watmough NJ, Frerman FE (2010) The electron transfer flavoprotein: ubiquinone oxidoreductases. Biochim Biophys Acta 1797: 1910–1916. doi: 10.1016/j.bbabio.2010.10.007 20937244 10.1016/j.bbabio.2010.10.007

[60] Hanukoglu I, Rapoport R, Weiner L, Sklan D (1993) Electron leakage from the mitochondrial NADPH-adrenodoxin reductase-adrenodoxin-P450scc (cholesterol side chain cleavage) system. Arch Biochem Biophys 305: 489–498. doi: 10.1006/abbi.1993.1452 8396893 10.1006/abbi.1993.1452

[61] Fieschi F, Niviere V, Frier C, Decout JL, Fontecave M (1995) The mechanism and substrate specificity of the NADPH:flavin oxidoreductase from Escherichia coli. J Biol Chem 270: 30392–30400. 8530465 10.1074/jbc.270.51.30392

[62] Fontecave M, Coves J, Pierre JL (1994) Ferric reductases or flavin reductases? Biometals 7: 3–8. 8118169 10.1007/BF00205187

[63] Lemire BD (2015) Evolution of FOXRED1, an FAD-dependent oxidoreductase necessary for NADH:ubiquinone oxidoreductase (Complex I) assembly. Biochim Biophys Acta 1847: 451–457. doi: 10.1016/j.bbabio.2015.01.014 25681241 10.1016/j.bbabio.2015.01.014

[64] Darlyuk I, Goldman A, Roberts SC, Ullman B, Rentsch D, Zilberstein D (2009) Arginine homeostasis and transport in the human pathogen Leishmania donovani. J Biol Chem 284: 19800–19807. doi: 10.1074/jbc.M901066200 19439418 10.1074/jbc.M901066200 PMC2740405

[65] Boitz JM, Gilroy CA, Olenyik TD, Paradis D, Perdeh J, Dearman K, et al (2017) Arginase Is Essential for Survival of Leishmania donovani Promastigotes but Not Intracellular Amastigotes. Infect Immun 85. 10.1128/IAI.00554-16 PMC5203656 27795357

[66] Roberts SC, Tancer MJ, Polinsky MR, Gibson KM, Heby O, Ullman B (2004) Arginase plays a pivotal role in polyamine precursor metabolism in Leishmania. Characterization of gene deletion mutants. J Biol Chem 279: 23668–23678. doi: 10.1074/jbc.M402042200 15023992 10.1074/jbc.M402042200

[67] Reguera RM, Balana-Fouce R, Showalter M, Hickerson S, Beverley SM (2009) Leishmania major lacking arginase (ARG) are auxotrophic for polyamines but retain infectivity to susceptible BALB/c mice. Mol Biochem Parasitol 165: 48–56. doi: 10.1016/j.molbiopara.2009.01.001 19393161 10.1016/j.molbiopara.2009.01.001 PMC2735255

[68] Gaur U, Roberts SC, Dalvi RP, Corraliza I, Ullman B, Wilson ME (2007) An effect of parasite-encoded arginase on the outcome of murine cutaneous leishmaniasis. J Immunol 179: 8446–8453. 18056391 10.4049/jimmunol.179.12.8446

[69] Muleme HM, Reguera RM, Berard A, Azinwi R, Jia P, Okwor IB, et al (2009) Infection with arginase-deficient Leishmania major reveals a parasite number-dependent and cytokine-independent regulation of host cellular arginase activity and disease pathogenesis. J Immunol 183: 8068–8076. doi: 10.4049/jimmunol.0803979 19923451 10.4049/jimmunol.0803979 PMC2800308

[70] Aoki JI, Muxel SM, Zampieri RA, Acuna SM, Fernandes JC, Vanderlinde RH, et al (2017) L-arginine availability and arginase activity: characterization of amino acid permease 3 in Leishmania amazonensis. PLoS Negl Trop Dis. 10.1371/journal.pntd.0006025 PMC5693463 29073150

[71] Mandal G, Wyllie S, Singh N, Sundar S, Fairlamb AH, Chatterjee M (2007) Increased levels of thiols protect antimony unresponsive Leishmania donovani field isolates against reactive oxygen species generated by trivalent antimony. Parasitology 134: 1679–1687. doi: 10.1017/S0031182007003150 17612420 10.1017/S0031182007003150 PMC3409873

[72] Adhuna A, Saltora P, Bhatnagar R (2000) Nitric oxide induced expression of stress proteins in virulent and avirulent promastigotes of Leishmania donovani. Immunol Lett 71: 171–176. 10722869 10.1016/s0165-2478(00)00158-9

[73] Salvati L, Mattu M, Colasanti M, Scalone A, Venturini G, Gradoni L, et al (2001) NO donors inhibit Leishmania infantum cysteine proteinase activity. Biochim Biophys Acta 1545: 357–366. 11342060 10.1016/s0167-4838(00)00297-1

[74] Holzmuller P, Hide M, Sereno D, Lemesre JL (2006) Leishmania infantum amastigotes resistant to nitric oxide cytotoxicity: Impact on in vitro parasite developmental cycle and metabolic enzyme activities. Infect Genet Evol 6: 187–197. doi: 10.1016/j.meegid.2005.03.003 15905133 10.1016/j.meegid.2005.03.003

[75] Mauel J, Ransijn A (1997) Leishmania spp.: mechanisms of toxicity of nitrogen oxidation products. Exp Parasitol 87: 98–111. doi: 10.1006/expr.1997.4205 9326885 10.1006/expr.1997.4205

