# Supplementary figures and images for: Arginase expression modulates nitric oxide production in Leishmania (Leishmania) amazonensis
Source: PLoS One. 2017 Nov 14;12(11):e0187186. doi: 10.1371/journal.pone.0187186 (PMC5685479; doi:10.1371/journal.pone.0187186)

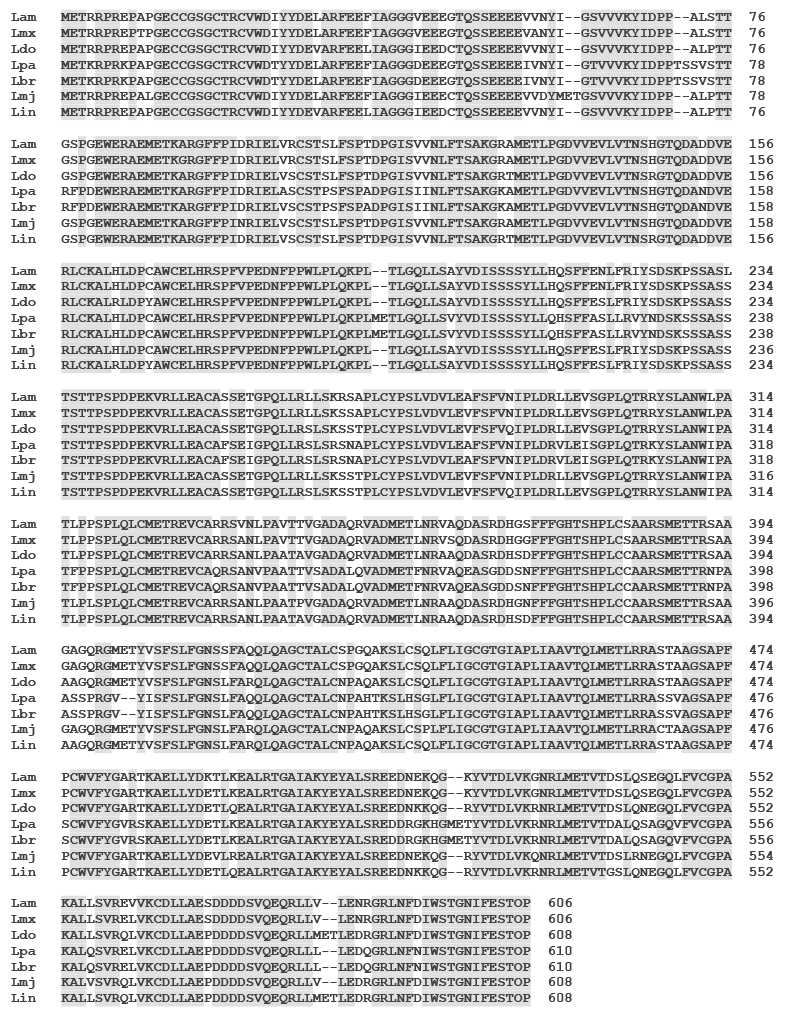

Supplement: S1 Fig — Multiple alignment of the amino acid sequence of the nitric oxide synthase-like NOS-like of. L. amazonensis (Lam), L. mexicana (Lmx), L. donovani (Ldo), L. panamensis (Lpa), L. braziliensis (Lbr), L. major (Lmj) and L. infantum. The identical amino acids are highlighted in gray. The alignment was performed based on NCBI multiple alignment tool. (TIF) [file pone.0187186.s001.tif]

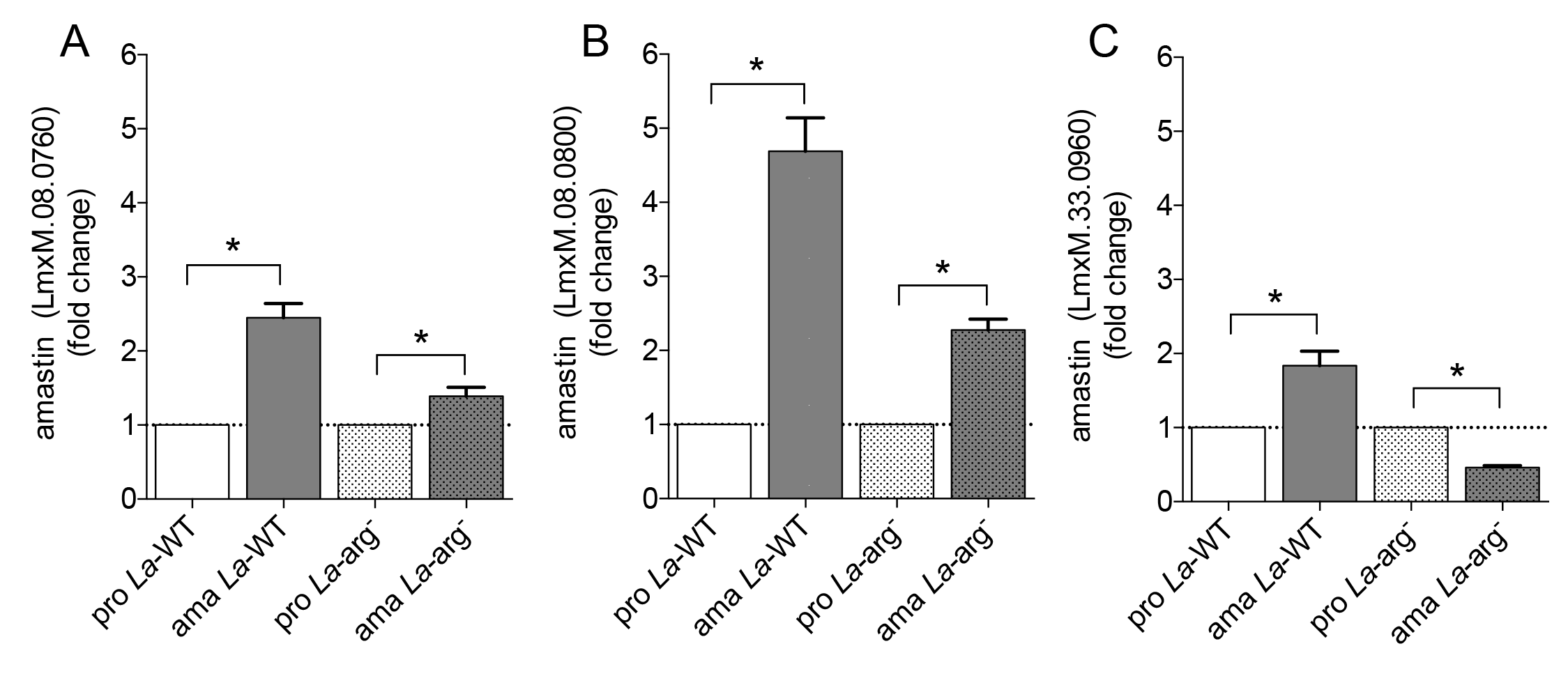

Supplement: S3 Fig — (A) The amastins LmxM.08.00760 (A), LmxM.0800800 (B) and LmxM.33.0960 (C) mRNA levels were based on quantification of the target and were normalized by gapdh expression in promastigotes (pro) and amastigotes (ama) of La-WT and La-arg-. The values are the mean ± SEM of three independent biological replicates (n = 4–6). (*) p < 0.05 (TIF) [file pone.0187186.s003.tif]
